# A novel role of Zebrafish TMEM33 in negative regulation of interferon production by two distinct mechanisms

**DOI:** 10.1371/journal.ppat.1009317

**Published:** 2021-02-18

**Authors:** Long-Feng Lu, Can Zhang, Zhuo-Cong Li, Xiao-Yu Zhou, Jing-Yu Jiang, Dan-Dan Chen, Yong-An Zhang, Feng Xiong, Fang Zhou, Shun Li

**Affiliations:** 1 Institute of Hydrobiology, Chinese Academy of Sciences, Wuhan, China; 2 University of Chinese Academy of Sciences, Beijing, China; 3 College of Fisheries and Life Science, Dalian Ocean University, Dalian, China; 4 State Key Laboratory of Agricultural Microbiology, College of Fisheries, Huazhong Agricultural University, Wuhan, China; Division of Clinical Research, UNITED STATES

## Abstract

The transmembrane protein 33 (TMEM33) was originally identified as an endoplasmic reticulum (ER) protein that influences the tubular structure of the ER and modulates intracellular calcium homeostasis. However, the role of TMEM33 in antiviral immunity in vertebrates has not been elucidated. In this article, we demonstrate that zebrafish TMEM33 is a negative regulator of virus-triggered interferon (IFN) induction via two mechanisms: mitochondrial antiviral signaling protein (MAVS) ubiquitination and a decrease in the kinase activity of TANK binding kinase 1 (TBK1). Upon stimulation with viral components, *tmem33* was remarkably upregulated in the zebrafish liver cell line. The IFNφ1 promoter (IFNφ1pro) activity and mRNA level induced by retinoic acid-inducible gene (RIG)-I-like receptors (RLRs) were significantly inhibited by TMEM33. Knockdown of TMEM33 increased host *ifn* transcription. Subsequently, we found that TMEM33 was colocalized in the ER and interacted with the RLR cascades, whereas MAVS was degraded by TMEM33 during the K48-linked ubiquitination. On the other hand, TMEM33 reduced the phosphorylation of mediator of IFN regulatory factor 3 (IRF3) activation (MITA)/IRF3 by acting as a decoy substrate of TBK1, which was also phosphorylated. A functional domain assay revealed that the N-terminal transmembrane domain 1 (TM1) and TM2 regions of TMEM33 were necessary for IFN suppression. Finally, TMEM33 significantly attenuated the host cellular antiviral capacity by blocking the IFN response. Taken together, our findings provide insight into the different mechanisms employed by TMEM33 in cellular IFN-mediated antiviral process.

## Author summary

The water environment of fish enhances the chance of aquatic viral infection which leads to host IFN production, thus fish IFN as a critical antiviral role need to be modulated precisely to avoid adverse pathogenic effects, however, the IFN expression balance in fish is still unclear. In this study, we identify that zebrafish transmembrane protein 33 (TMEM33) negatively regulates IFN production by two distinct mechanisms: TMEM33 interacted with and degraded MAVS through K48-linked ubiquitination; TMEM33 acted as a decoy substrate for cellular TBK1 and reduced the phosphorylation of MITA/IRF3. Our findings reveal the role for fish TMEM33 in regulating cellular immune responses and expand our knowledge of the IFN negative regulatory mechanisms.

## Introduction

The innate immune system constitutes the first line of host cell defense against invading pathogens [1]. Pattern-recognition receptors (PRRs), such as retinoic acid-inducible gene 1 (RIG-I)-like receptors (RLRs), Toll-like receptors (TLRs), and nucleotide-binding oligomerization domain (NOD)-like receptors (NLRs), recognize structurally conserved pathogen-associated molecular patterns (PAMPs) and trigger a series of signaling events that lead to the induction of cytokines including interferons (IFNs) [2, 3]. The RLR family comprises three members: RIG-I, melanoma differentiation-associated factor 5 (MDA5), and laboratory of genetics and physiology 2 (LGP2) [3, 4]. Upon activation, both RIG-I and MDA5 interact with downstream mitochondrial antiviral signaling protein (MAVS, also known as VISA, IPS-1, and Cardif) [5–8] and mitochondrial- and endoplasmic reticulum (ER)-localized adapter protein mediator of IFN regulatory factor 3 (IRF3) activation (MITA) (also called STING, MPYS, and ERIS) [9–12]. They then recruit the downstream kinase TANK-binding kinase 1 (TBK1), which is responsible for phosphorylating and activating IRF3/7, leading to the transcription of IFNs and other cytokines [13–15]. To date, the exogenous RNA sensors of RLRs have been identified in fish [16–20], and the signal transduction of downstream cascades for MAVS, MITA, TBK1, and IRF3/7 have also been reported [21–27]. As examples, both the full-length and N-terminal caspase activation and recruitment domains (CARDs) of fish RIG-I induced IFN and IFN-stimulated genes (ISGs), resulting in a strong antiviral state that deters the infection of viruses [17]. Similarly, MAVS and TBK1 induce the constitutive expression of IFN and ISGs, conferring on fish cells protection against virus infection [21, 25]. These observations suggest that the RLR pathway is highly conserved from teleost fish to mammals [28].

Though IFN production is necessary for the host to defend against viruses, uncontrolled expression of IFN is hazardous to the host and results in autoimmune diseases [29]. Usually, IFN-positive regulators promote the antiviral immune response to control and clear viral infections and negative regulators are also necessary to dampen inflammatory responses and prevent spontaneous autoimmunity [30, 31]. During the host antivirus response, the RLR pathway is tightly regulated to achieve an orchestrated response aimed at maximizing antiviral immunity and preventing unwanted or inappropriate cellular responses [30]. MAVS and TBK1 are critical molecules in RLR-mediated signaling pathways and play vital roles in facilitating the immune responses to viral pathogens [7, 32, 33]. Therefore, their activities must be tightly controlled to ensure a proper innate immune homeostasis [34]. In mammals, for MAVS, the E3-ubiquitin (Ub) ligase smad ubiquitin regulatory factor (Smurf) 2 associates with MAVS to promote K48-linked ubiquitination and degradation, resulting in the reduction of IFN activity [35]. The enhancer of zeste homolog 2 (EZH2) interacts with the CARD region of MAVS and prevents RIG-I-MAVS interactions, inhibiting IFN induction [36]. With regard to TBK1, TNF receptor-associated factor (TRAF3)-interacting protein 3 (TRAF3IP3) interacts with and targets TBK1 for K48-linked ubiquitination, leading to the degradation of TBK1 and the restriction of IFN expression [37]. DExD/H-box RNA helicase 19 (DDX19) negatively regulates IFN production by impeding the formation of TBK1/inhibitor-κB kinase ε (IKKε)-IRF3 complexes and promoting TBK1 and IKKε degradation [38]. These findings demonstrate that the cascades of the RLR axis are precisely regulated for IFN activation in higher vertebrates.

In fish, IFN is likewise the pivotal cytokine in the defense against viral infections [39]. For IFN expression balance, several fish proteins have been identified as negative regulators that target the key molecules of RLR signaling to block IFN production. For instance, in zebrafish, the protein inhibitor of activated signal transducer and activator of transcription (PIAS) 4a plays a vital role in the suppression of MAVS-induced IFN activation [40]. IRF10 suppresses IFN expression by binding to the IFN-stimulated response element (ISRE) motif of the IFN promoter and weakening MITA activation [41]. TBK1 spliced isoforms block IFN induction by disrupting TBK1-IRF3 interactions and inhibiting IRF3 phosphorylation [42]. Forkhead box O (FOXO) 3b negatively regulates the IFN response by preventing the activation of IRF3 and IRF7 [43]. Though evidence that IFN production is negatively modulated by the host has been found in fish, the fish IFN brake system remains mysterious.

In mammals, transmembrane protein 33 (TMEM33), which contains three transmembrane domains (TMs) in the N-terminal, localizes to the ER and participates in the regulation of the tubular ER structure through the modulation of reticulon activity [44]. TMEM33 is a newly identified ER stress-inducible molecule that regulates the unfolded protein response (UPR) signaling and is a key modulator of intracellular calcium homeostasis in renal tubular epithelial cells through interactions with polycystin-2 (PC2) at the ER [45, 46]. TMEM33 is conserved from zebrafish to humans, with 78% homology. Recent reports have shown that TMEM33 is required for vascular endothelial growth factor (VEGF)-mediated calcium oscillations and angiogenesis in both human and zebrafish endothelial cells (ECs) [47]. To date, the roles of TMEM33 in the host immune processes of both fish and mammals have not been reported.

In this study, zebrafish TMEM33 was identified as a IFN negative regulator with two mechanisms: it promotes the K48-linked Ub-proteasomal degradation of MAVS and acts as a competitive substrate of TBK1 to reduce MITA/IRF3 phosphorylation, leading to the inhibition of IFN induction and cellular antiviral responses. Our findings reveal the distinct mechanisms through which TMEM33 suppresses IFN. These data may provide clues for understanding the specific immune regulatory mechanisms in fish and the underlying mechanisms of human TMEM33 in antiviral innate immunity.

## Results

### Zebrafish *tmem33* is upregulated by SVCV and poly I:C stimulation

**Fig 1:**
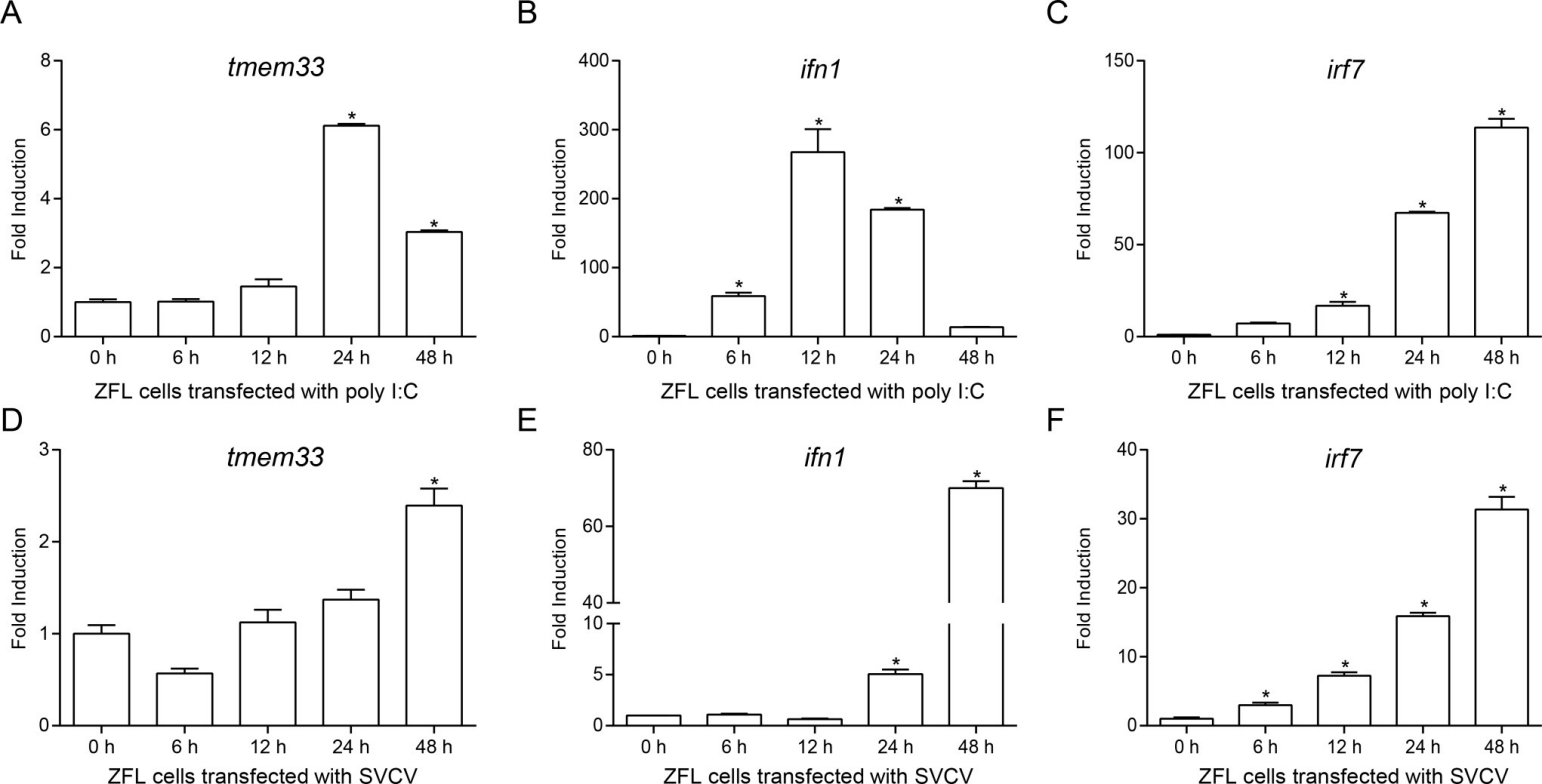
TMEM33 is stimulated by virus infection. (A-F) qPCR detection of the transcriptional levels of *tmem33*, *ifn1*, and *irf7* on stimulation. ZFL cells seeded on 6-well plates overnight and transfected with poly I:C (1 μg/ml) (A-C) or stimulated with SVCV (MOI = 1) (D-F). At the time points 6, 12, 24, and 48 h, total RNAs were extracted for further qPCR assays. *β-actin* was used as an internal control for normalization and the relative expression is represented as fold induction relative to the expression level in control cells (set to 1). Data were expressed as mean ± SEM, *n* = 3. Asterisks indicate significant differences from control (*, *p* < 0.05).

It has been reported that the fish RLR signaling pathway plays a critical role in IFN induction and cellular antiviral responses [48]. However, the negative regulatory mechanisms responsible for RLR-mediated IFN signaling remain poorly understood. Using Co-IP screening and mass spectrum analysis, one of the TMEM proteins, which was termed TMEM33, was screened. Afterward, the ORF of zebrafish TMEM33 was amplified from a cDNA library of ZFL cells stimulated with poly I:C to assess whether TMEM33 is involved in the antivirus process. To investigate the expression pattern of zebrafish TMEM33, qPCR assays were performed to detect *tmem33* transcripts in ZFL cells. As shown in Fig 1A, the mRNA of *tmem33* was induced at 6 h after poly I:C transfection and reached a peak at 24 h. *ifn1* and *irf7*, known to be antiviral genes, were also upregulated during the induction (Fig 1B and 1C). Similar patterns in the expression of *tmem33*, *ifn1*, and *irf7* were also observed when the cells were infected with SVCV, indicating that *tmem33* mRNA increased under virus infection, similar to *ifn1* and *irf7* (Fig 1D–1F). These results suggest that zebrafish TMEM33 is induced to respond to virus infection and thus is likely involved in the antiviral response process.

### Zebrafish TMEM33 blocks poly I:C and SVCV-induced IFN activation

**Fig 2:**
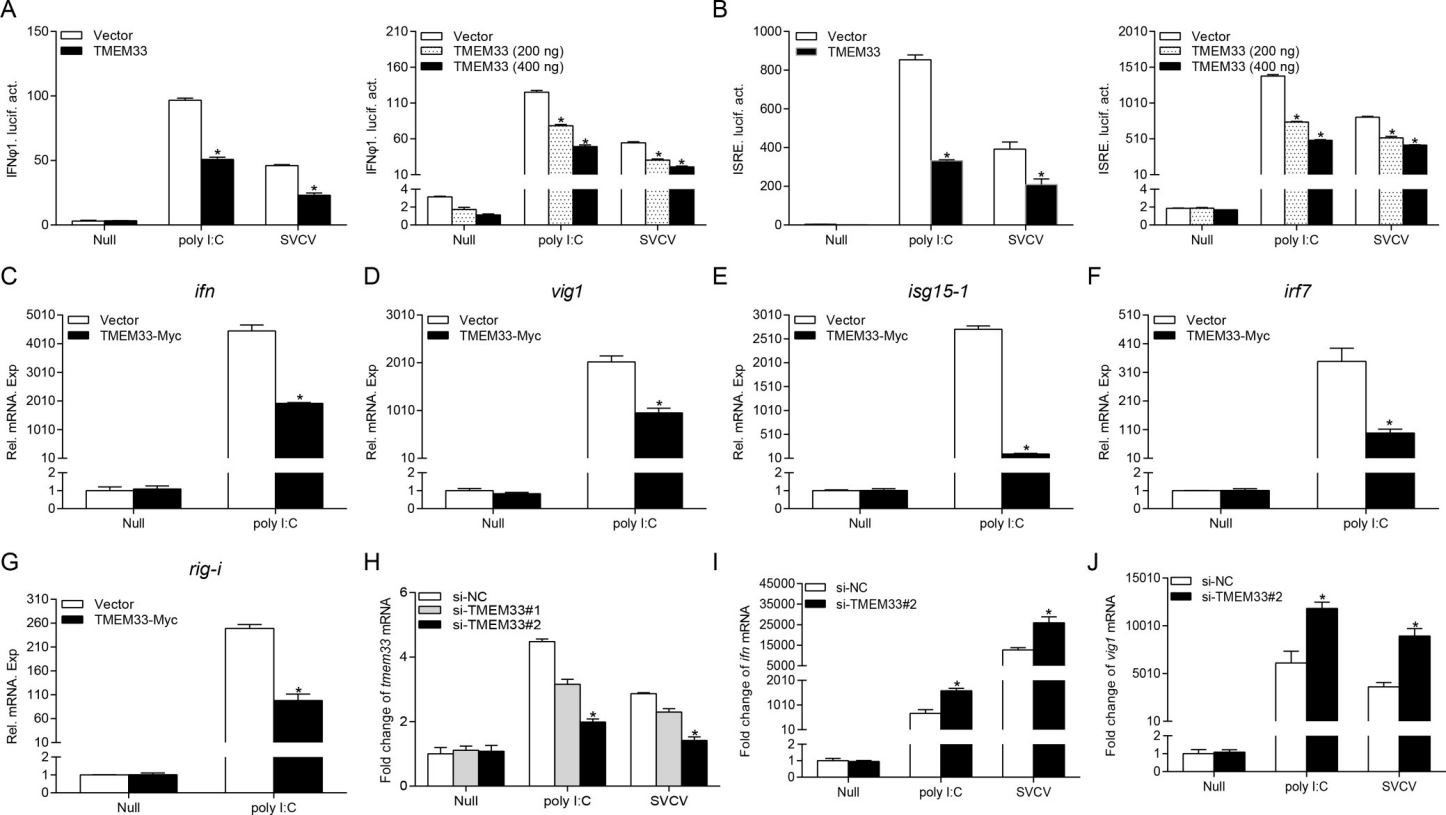
Inhibition of IFN activation by overexpression of TMEM33. (A and B) Overexpression of TMEM33 suppresses poly I:C/SVCV-induced IFNφ1pro/ISRE-Luc activation and displays a dose-dependent manner. EPC cells were seeded in 24-well plates and transfected the next day with 250 ng IFNφ1pro-Luc (A) or ISRE-Luc (B) and 25 ng pRL-TK, plus pcDNA3.1-TMEM33 (250 ng or 200/400 ng) or pcDNA3.1(+) (control vector). At 24 h post-transfection, cells were untreated (null) or transfected with poly I:C (1 μg/ml) or treated with SVCV (MOI = 1). Luciferase activities were monitored at 24 h after stimulation. The promoter activity is presented as relative light units (RLU) normalized to *Renilla* luciferase activity. (C-G) Overexpression of TMEM33 inhibits the expression of *ifn* (C), *vig1* (D), *isg15-1* (E), *irf7* (F), and *rig-i* (G) induced by poly I:C in EPC cells. EPC cells seeded in 6-well plates overnight were transfected with 2 μg TMEM33-Myc or empty vector and transfected with poly I:C at 24 h post-transfection. At 24 h after stimulation, total RNAs were extracted to examine the mRNA levels of cellular *ifn*, *vig1*, *isg15-1*, *irf7*, and *rig-i*. (H) Effects of TMEM33 RNAi on the expression of endogenous TMEM33. EPC cells were seeded in 6-well plates overnight and transfected with 100 nM si-TMEM33#1, si-TMEM33#2, or si-NC (negative control). At 24 h post-transfection, the cells were transfected with poly I:C or treated with SVCV (MOI = 1). At 24 h post-stimulation, total RNAs were extracted to examine the transcriptional levels of TMEM33. (I and J) Effects of TMEM33 RNAi on the poly I:C/SVCV-induced *epc ifn* and *epc vig1* transcription. EPC cells were seeded in 6-well plates and transfected with 100 nM si-NC or si-TMEM33#2. At 24 h post-transfection, cells were untreated or transfected with poly I:C or treated with SVCV for 24 h before qPCR analysis was performed. The relative transcriptional levels were normalized to the transcription of *β-actin* and represented as fold induction relative to the transcriptional level in the control cells, which was set to 1. Data were expressed as mean ± SEM, *n* = 3. Asterisks indicate significant differences from control (*, *p* < 0.05).

Previous studies have demonstrated that zebrafish IFNφ1 plays a significant role in innate antiviral responses [49]. Therefore, the promoter of IFNφ1 (IFNφ1pro) was used in subsequent assays in EPC cells with higher transfection efficiency. As shown in Fig 2A, poly I:C stimulation or SVCV infection induced the activation of IFNφ1pro and this activation was significantly inhibited by TMEM33. The ISRE motif is considered the binding site for ISGs responding to transcriptional factors. The activation of ISRE also decreased in the TMEM33 group compared to that in the control group under stimulation, and both IFNφ1pro and ISRE were suppressed by TMEM33 in a dose-dependent manner (Fig 2B). Besides the promoter activities, the transcripts of the host IFN response systems were also monitored. TMEM33 consistently dampened the mRNA level of *ifn* when stimulated and this inhibition was also observed with the other ISGs (Fig 2C–2G). Next, to investigate the function of endogenous TMEM33 in IFN production, two EPC TMEM33-specific siRNAs were designed and employed. In the *tmem33* knockdown efficiency assay, si-TMEM33#2 displayed a greater capability to knockdown the expression of *tmem33* under stimulation and was therefore selected for subsequent experiments (Fig 2H). In qPCR assays, the production of *epc ifn* triggered by poly I:C or SVCV increased after TMEM33 knockdown and the expression of *epc vig1* was also upregulated after treatment with TMEM33 siRNA (Fig 2I and 2J). Collectively, these data demonstrate that TMEM33 inhibits IFN activation induced by the virus.

### Zebrafish TMEM33 inhibits the cellular antiviral response against SVCV infection

**Fig 3:**
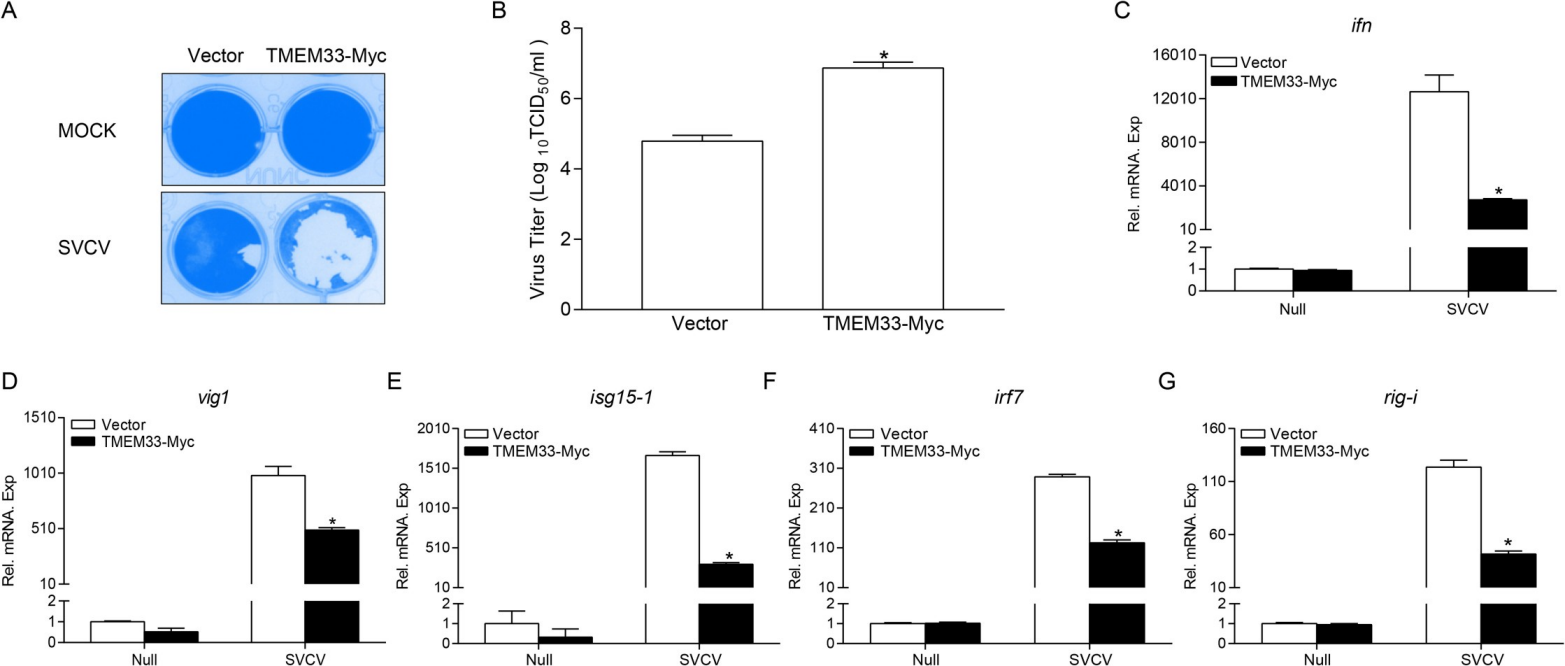
Zebrafish TMEM33 attenuates the cellular antiviral response. (A and B) Enhance of virus replication by overexpression of TMEM33. EPC cells seeded in 24-well plates overnight were transfected with 0.5 μg of TMEM33-Myc or empty vector. At 24 h post-transfection, cells were infected with SVCV (MOI = 0.001) for 48 h. Then, cells were fixed with 4% PFA and stained with 1% crystal violet (A). Culture supernatants from the cells infected with SVCV were collected, and the viral titer was measured according to the method of Reed and Muench (B). (C-G) Overexpression of TMEM33 inhibits the expression of *ifn* (C), *vig1* (D), *isg15-1* (E), *irf7* (F), and *rig-i* (G) induced by SVCV infection. EPC cells seeded in 6-well plates overnight were transfected with 2 μg of TMEM33-Myc or empty vector and infected with SVCV (MOI = 1) at 24 h post-transfection. At 24 h after infection, total RNAs were extracted to examine the mRNA levels of cellular *ifn*, *vig1*, *isg15-1*, *irf7*, and *rig-i*. The relative transcriptional levels were normalized to the transcriptional level of the *β-actin* gene and were represented as fold induction relative to the transcriptional level in the control cells, which was set to 1. Data were expressed as mean ± SEM, n = 3. Asterisks indicate significant differences from control values (*, *p* < 0.05).

To identify the general biological role of TMEM33 in the cellular antiviral capacity, EPC cells were transfected with TMEM33 and infected with SVCV. At 48 h post-infection, substantially more CPE was observed in the TMEM33 group compared with that in the control cells (Fig 3A). The SVCV titer in each group further confirmed, it was significantly increased (110-fold) in the supernatant of TMEM33-overexpressed cells compared with the level in the control cells (Fig 3B). In addition, the expression of *ifn* and related ISGs induced by viral infection was analyzed with qPCR assays. As shown in Fig 3C–3G, TMEM33 decreased the downstream ISGs to different degrees. These results suggest that TMEM33 negatively regulates the cellular antiviral response by weakening the expression of key antiviral genes and facilitating the proliferation of SVCV.

### Zebrafish TMEM33 suppresses IFN induction by reducing RLR signaling pathway activity

**Fig 4:**
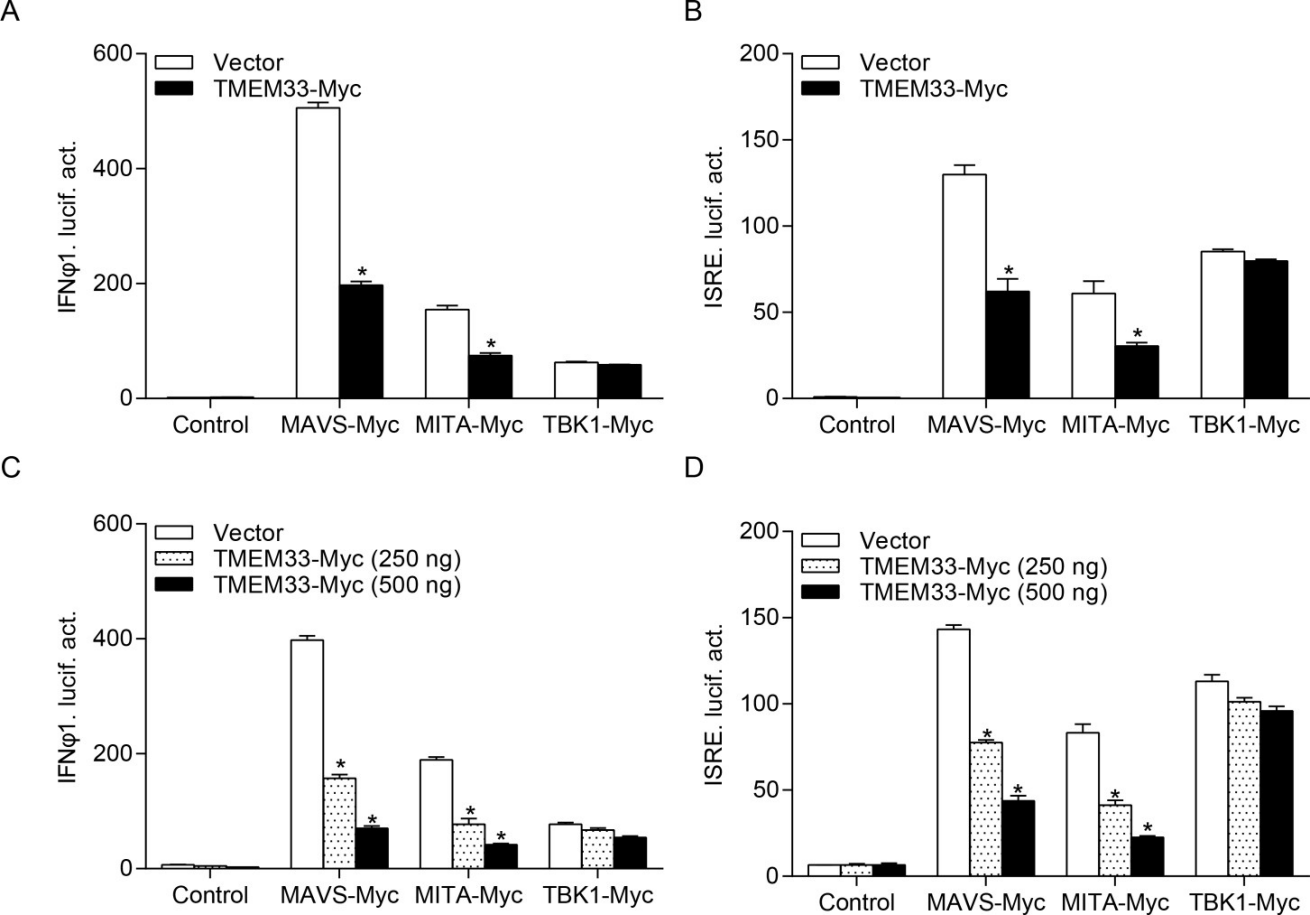
Zebrafish TMEM33 blocks IFNφ1/ISRE activation mediated by MAVS or MITA in a dose-dependent manner. (A and B) EPC cells were seeded into 24-well plates overnight and co-transfected with MAVS-, MITA-, or TBK1-expressing plasmid and empty vector or TMEM33-Myc (250 ng or 250/500 ng), plus IFNφ1pro-Luc (A and C) or ISRE-Luc (B and D) at the ratio of 1:1:1. pRL-TK was used as a control. At 24 h post-transfection, cells were lysed for luciferase activity detection. Data were expressed as mean ± SEM, *n* = 3. Asterisks indicate significant differences from control (*, *p* < 0.05).

A few reports have indicated that fish RLR signaling pathways are crucial for IFN production as in mammals [41]. We subsequently investigated whether the capacity of the RLRs to reduce IFN expression is modulated by TMEM33. As shown in Fig 4A and 4B, IFNφ1pro and ISRE were induced by the RLR factors, whereas the activation driven by MAVS and MITA was suppressed by TMEM33, and TBK1 was not affected. Subsequent analysis demonstrated that the inhibitory activity of TMEM33 was dose-dependent (Fig 4C and 4D). Since TBK1 is the downstream factor of MAVS and MITA and overexpression of TBK1 rescued the decline of IFN, we speculated that MAVS or MITA and TBK1 were the target(s) of TMEM33. These results suggest that TMEM33 disrupts the induction of IFN by MAVS but has little effect on the overexpression of TBK1.

### Zebrafish TMEM33 interacts with RLRs and is localized at the ER

**Fig 5:**
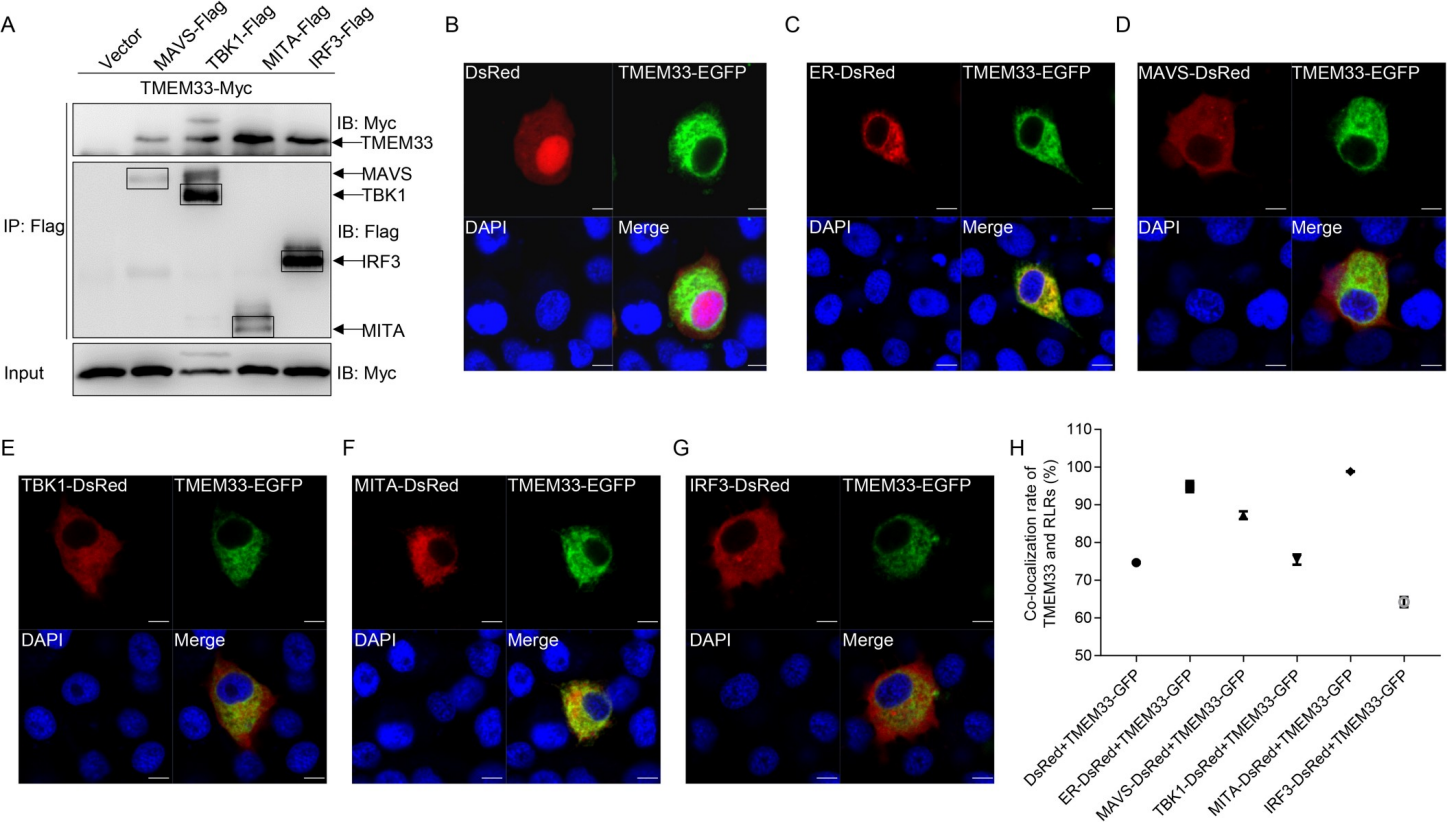
Zebrafish TMEM33 interacts with RLRs and localizes at the ER. (A) TMEM33 interacts with MAVS, MITA, TBK1, and IRF3. EPC cells seeded in 10 cm^2^ dishes were transfected with the indicated plasmids (5 μg each). After 24 h, cell lysates were immunoprecipitated (IP) with anti-Flag affinity gel. Then the immunoprecipitates and WCLs were analyzed by IB with the anti-Myc and anti-Flag Abs, respectively. (B and C) TMEM33 is localized at the ER. EPC cells seeded onto microscopy cover glass in 6-well plates were co-transfected with 1 μg TMEM33-EGFP and 1 μg empty vector (B) or ER-DsRed (C). After 24 h, the cells were fixed and subjected to confocal microscopy analysis. Green signals represent overexpressed TMEM33, and blue staining indicates the nucleus region. The yellow staining in the merged image indicates that TMEM33 is localized at the ER. (D-G) TMEM33 colocalizes with MAVS and MITA. EPC cells were plated onto coverslips in 6-well plates and co-transfected with 1 μg TMEM33-EGFP and 1 μg MAVS-DsRed (D), TBK1-DsRed (E), MITA-DsRed (F), or IRF3-DsRed (G). After 24 h, the cells were fixed and observed by confocal microscopy. Green signals represent overexpressed TMEM33. Red signals represent overexpressed MAVS, TBK1, MITA, or IRF3, and blue staining indicates the nucleus region. The yellow staining in the merged image indicates the colocalization of TMEM33 and MAVS or MITA (original magnification 63×; oil immersion objective). Scale bar, 10 μm. (H) The colocalization rates of B-G were performed by LAS AF Lite. All experiments were repeated for at least three times with similar results.

Given that TMEM33 negatively regulates the function of RLR molecules in activating IFN expression, the relationships between TMEM33 and RLRs were clarified using Co-IP and confocal microscopy assays. First, the interactions between TMEM33 and RLR signaling molecules were examined. EPC cells were co-transfected with TMEM33-Myc and Flag-tagged RLRs, including MAVS, MITA, TBK1, and IRF3. The results revealed that all the anti-Flag Ab-immunoprecipitated protein complexes containing RLR factors were recognized by the anti-Myc Ab, suggesting that TMEM33 associates with these RLR proteins. Interestingly, a high molecular band was observed in the TBK1 group, indicating that TMEM33 may be phosphorylated by TBK1 (Fig 5A). Furthermore, the subcellular colocalizations of TMEM33 with RLRs were investigated. Given that zebrafish TMEM33 harbors three predicted TMs within its N terminus, the subcellular localization of TMEM33 was elucidated first. After co-transfection with TMEM33-enhanced green fluorescent protein (EGFP) and an empty vector (DsRed) or ER-DsRed (ER marker), confocal microscopy analysis demonstrated that the green signals of TMEM33 were entirely overlapped with the red signals of the ER marker, suggesting that fish TMEM33 is located at the ER membrane (Fig 5B and 5C). In addition, the green signals from TMEM33 were uniformly overlapped with the red signals from MITA and partly colocalized with the red signals from MAVS, TBK1, and IRF3 (Fig 5D–5H). Taken together, these results suggest that TMEM33 associates with the RLR factors and is localized to the ER region.

**Fig 6:**
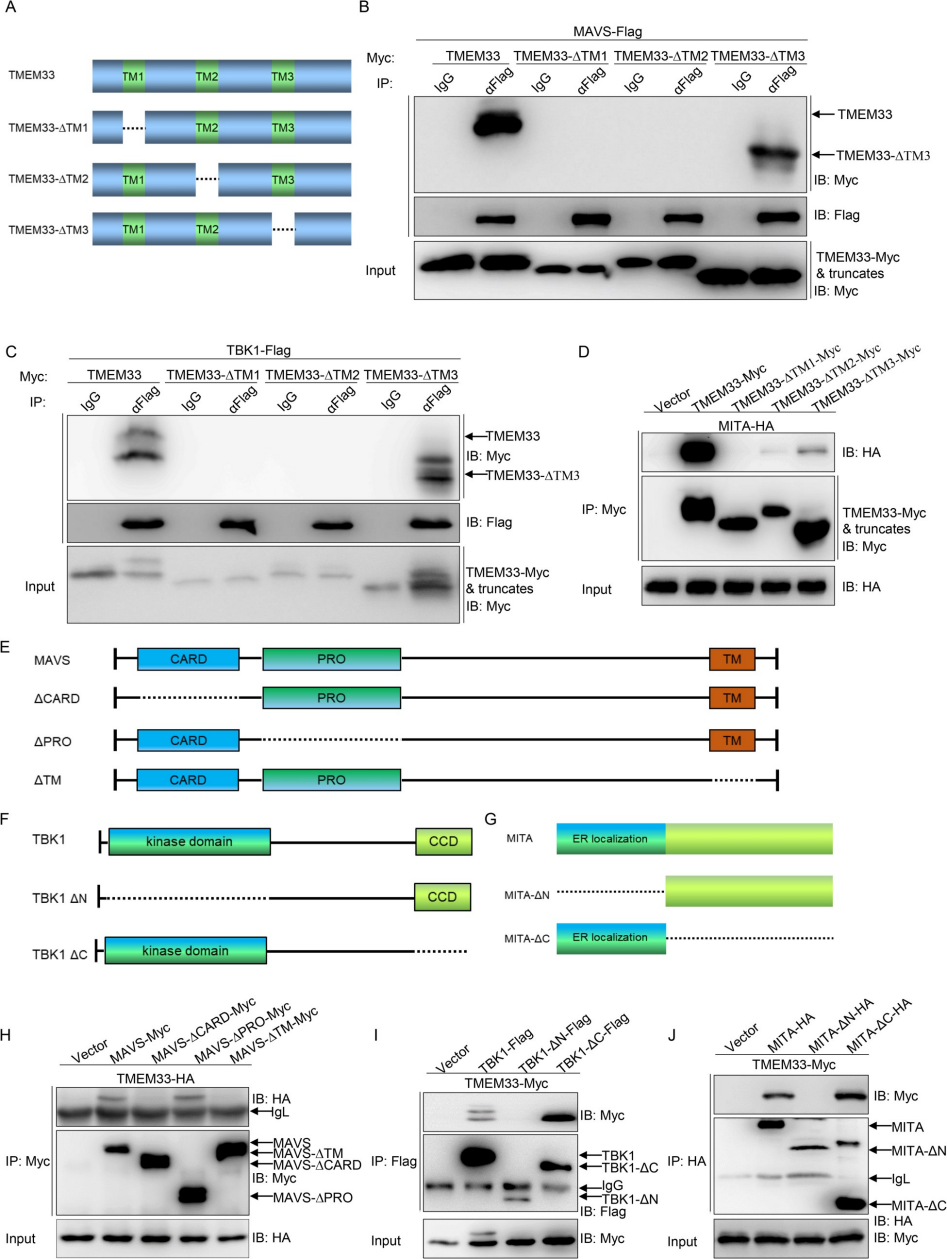
Domain mapping of the TMEM33-MAVS, TMEM33-TBK1, and TMEM33-MITA interaction. (A) Schematic representation of full-length TMEM33 and its mutants. (B-D) The first and the second TM regions of TMEM33 are necessary for its association with MAVS, TBK1, and MITA. HEK 293T cells seeded in 10 cm^2^ dishes were co-transfected with the indicated plasmids (5 μg each). After 24 h, cell lysates were immunoprecipitated (IP) with anti-Flag affinity gel (B and C) or anti-Myc affinity gel (D). Then the immunoprecipitates and WCLs were analyzed by IB with the indicated Abs. (E-G) Diagrammatic representations of full-length MAVS/TBK1/MITA and deletion mutants in this study. (H) The N-terminal CARD domain and C-terminal TM domain of MAVS are responsible to its interaction with TMEM33. The experiments were performed similarly as described above for panel B. (I) The N-terminal kinase domain of TBK1 is essential to its interaction with TMEM33. The experiments were performed similarly as described above for panel B. (J) TMEM33 associates with MITA via its N terminus. The experiments were performed similarly as described above for panel B. All experiments were repeated for at least three times with similar results.

Next, a series of TMEM33 truncations were generated and used to explore the ability of TMEM33 to interact with MAVS, MITA, and TBK1. Domain mapping analysis and Co-IP assays indicated that the first and second TM regions of TMEM33 were responsible for its association with MAVS, MITA, and TBK1 (Fig 6A–6D). Accordingly, further domain mapping analysis was performed to determine the domains of MAVS, MITA, or TBK1 which are required for their interaction with TMEM33. As shown in Fig 6E and 6H, TMEM33 interacted with the full-length MAVS as well as truncations of MAVS that contained both the N-terminal CARD domain and the C-terminal TM domain. For TBK1, two truncations were generated: TBK1-ΔN, lacking the N-terminal kinase domain, and TBK1-ΔC, lacking the C-terminal coiled-coil domain (Fig 6F). As shown in Fig 6I, consistent with wild-type TBK1, TBK1-ΔC bound to TMEM33, but the association was abrogated in TBK1-ΔN group. We also found that the N-terminal ER localization domain of MITA was required for TMEM33 association (Fig 6G and 6J). Collectively, these data suggest that TMEM33 is localized at the ER and interacts with MAVS, TBK1, and MITA. The data also reveals the functional domains involved in the interactions between these molecules.

### Zebrafish TMEM33 promotes K48-linked ubiquitination and degradation of MAVS

**Fig 7:**
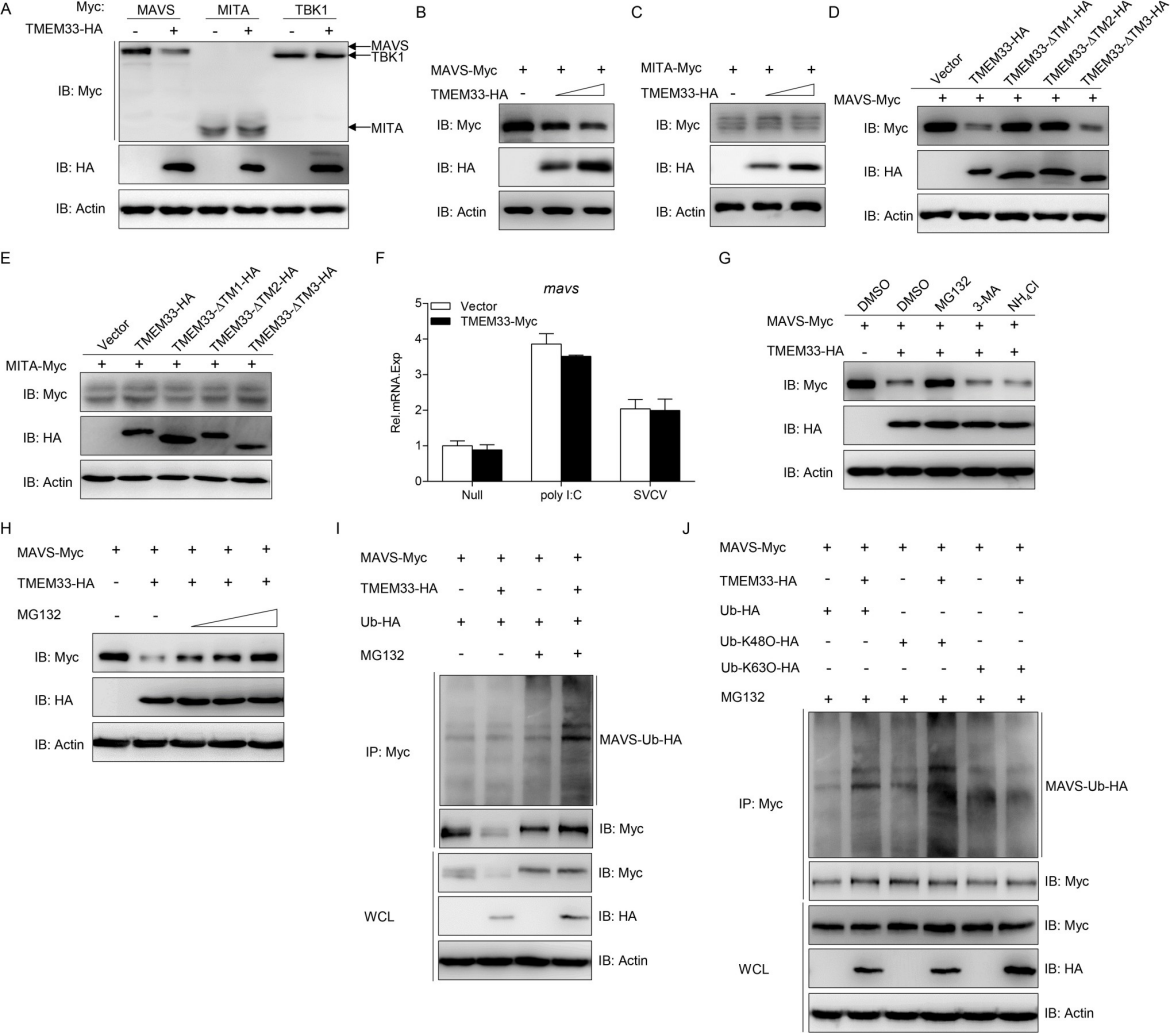
Zebrafish TMEM33 mediates ubiquitination of MAVS. (A-C) Overexpression of TMEM33 degrades MAVS in a dose-dependent manner. EPC cells were seeded in 6-well plates overnight and co-transfected with 1 μg TMEM33-HA and 1 μg empty vector, MAVS-Myc, MITA-Myc, or TBK1-Myc (A); TMEM33-HA (1 or 2 μg) and 1 μg MAVS-Myc (B) or MITA-Myc (C) for 24 h. The cell lysates were subjected to IB with anti-Myc, anti-HA, and anti-β-actin Abs. (D and E) TMEM33-ΔTM1 and TMEM33-ΔTM2 have no effect on the exogenous MAVS and MITA. EPC cells were seeded in 6-well plates overnight and transfected with the indicated plasmids (1 μg each) for 24 h. The cell lysates were subjected to IB with the indicated Abs. (F) TMEM33 has no influences on the poly I:C/SVCV-induced transcriptions of MAVS. EPC cells were transfected with 2 μg TMEM33-Myc or empty vector for 24 h, and then transfected with poly I:C or infected with SVCV (MOI = 1) for 24 h. Total RNAs were extracted to examine the mRNA level of *mavs* by qPCR. The relative transcriptional levels were normalized to the transcription of *β-actin* and represented as fold induction relative to the transcriptional level in the control cells, which was set to 1. Data were expressed as mean ± SEM, *n* = 3. (G) Effects of inhibitors on TMEM33-mediated destabilization of MAVS. EPC cells were seeded in 6-well plates overnight and co-transfected the indicted plasmids. At 18 h post-transfection, the cells were treated with DMSO, MG132 (20 μM), 3-MA (2 mM), or NH_4_Cl (20 mM) for 6 h. The cell lysates were subjected to IB with the indicated Abs. (H) TMEM33-induced MAVS degradation is rescued by MG132 in a dose-dependent manner. EPC cells were seeded in 6-well plates overnight and co-transfected the indicted plasmids. At 18 h post-transfection, the cells were treated with DMSO or MG132 (10, 20, or 40 μM) for 6 h. Then, the cells were harvested for IB with the indicated Abs. (I) TMEM33 promotes the ubiquitination of MAVS. EPC cells were transfected with 5 μg MAVS-Myc, 5 μg TMEM33-HA or empty vector, and 1 μg Ub-HA. At 18 h post-transfection, the cells were treated with DMSO or MG132 for 6 h. Cell lysates were IP with anti-Myc affinity gel. Then the immunoprecipitates and WCLs were analyzed by IB with the indicated Abs. (J) TMEM33 mediates K48-linked ubiquitination of MAVS *in vivo*. EPC cells were transfected with 5 μg MAVS-Myc, 5 μg TMEM33-HA or empty vector, and 1 μg Ub-HA, Ub-K48O-HA or Ub-K63O-HA. At 18 h post-transfection, the cells were treated with MG132 for 6 h. At 24 h post-transfection, cell lysates were IP with anti-Myc-affinity gel. Then the immunoprecipitates and WCLs were analyzed by IB with the indicated Abs. All experiments were repeated for at least three times with similar results.

To further elucidate the mechanism through which TMEM33 regulates the RLR factors, we first examined whether TMEM33 had any effect on the RLR molecules at the protein level. As shown in Fig 7A, MAVS was extremely reduced by the overexpression of TMEM33, but there was no obvious change in MITA and TBK1. In addition, the decline of MAVS mediated by TMEM33 was dose-dependent (Fig 7B). As a control, the abundance of MITA did not change according to the concentration of TMEM33 (Fig 7C). Consistently, analysis of the truncated mutants showed that MAVS was not affected by TMEM33-ΔTM1 and TMEM33-ΔTM2 (Fig 7D and 7E). In addition, to identify whether the decline of MAVS is regulated at the mRNA level, the transcription of MAVS was monitored in the presence or absence of poly I:C stimulation or SVCV infection. The results demonstrated that the mRNA of MAVS was not affected by TMEM33, indicating that TMEM33 negatively regulates MAVS expression at the protein level (Fig 7F). Next, the degradation mechanism of MAVS in this process was investigated. Protein degradation is mediated through three classical mechanisms, namely the ubiquitin-proteasome, autophagosome, and lysosomal pathways, and these pathways are disrupted by MG132, 3-MA, and NH_4_Cl, respectively. As shown in Fig 7G, TMEM33-mediated degradation of MAVS was significantly blocked by the proteasome inhibitor MG132 but not by 3-MA or NH_4_Cl. Moreover, the MAVS protein levels were gradually rescued as the concentration of MG132 increased, indicating that the degradation of MAVS by TMEM33 is proteasome-dependent (Fig 7H). Since ubiquitination is an important process during proteasome-dependent degradation, we further examined whether TMEM33-induced degradation of MAVS was due to ubiquitination. EPC cells were transfected with expression plasmids for MAVS-Myc, Ub-HA, and TMEM33-HA in the presence or absence of MG132. Following the immunoprecipitation of MAVS-Myc, immunoblot analysis revealed that TMEM33 potentiated the ubiquitination of MAVS (Fig 7I). Usually, K48-linked polyubiquitin chain modification leads to the target proteins for proteasome recognition and degradation, and K63-linked polyubiquitin chain modification enhances the stability of the target proteins [50, 51]. The pattern of MAVS ubiquitination through K48- or K63-linked ubiquitination was assessed. As shown in Fig 7J, TMEM33 promoted MAVS ubiquitination with wild-type Ub and Ub-K48 but not with Ub-K63. Collectively, these results suggest that TMEM33 induces the K48-linked Ub-proteasomal degradation of MAVS.

### Zebrafish TMEM33 inhibits MAVS-mediated cellular antiviral response

**Fig 8:**
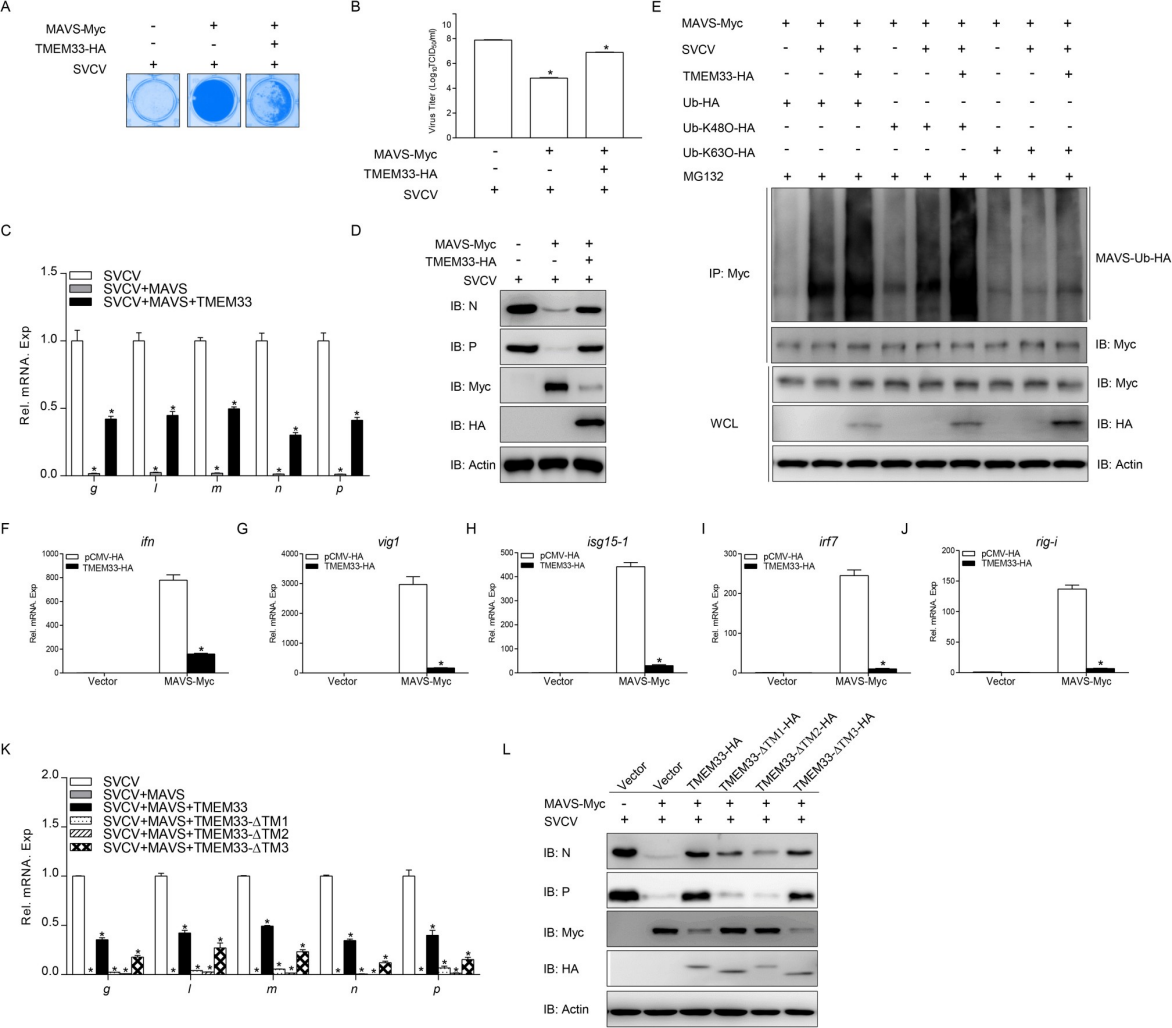
Zebrafish TMEM33 attenuates MAVS-induced cellular antiviral function. (A and B) Overexpression of TMEM33 restores MAVS-mediated decrease of viral titer. EPC cells seeded in 24-well plates overnight were transfected with 0.25 μg of MAVS-Myc and 0.25 μg of TMEM33-HA or empty vector. At 24 h post-transfection, cells were infected with SVCV (MOI = 0.001) for 48 h. Then, cells were fixed with 4% PFA and stained with 1% crystal violet (A). Culture supernatants from the cells infected with SVCV were collected, and the viral titer was measured according to the method of Reed and Muench (B). (C) EPC cells were seeded in 6-well plates overnight and then transfected with 2 μg of MAVS-Myc and 2 μg of TMEM33-HA or empty vector. At 24 h post-transfection, cells were infected with SVCV (MOI = 1). After 24 h-infection, total RNAs were extracted to examine the mRNA levels of cellular *g*, *l*, *m*, *n*, and *p*. The relative transcriptional levels were normalized to the transcriptional level of the *β-actin* gene and were represented as fold induction relative to the transcriptional level in the control cells, which was set to 1. Data were expressed as mean ± SEM, n = 3. Asterisks indicate significant differences from control values (*, *p* < 0.05). (D) The same samples were prepared similarly as described above for panel C. The lysates were detected by IB with the anti-N, anti-P, anti-Myc, anti-HA, and anti-β-actin Abs, respectively. (E) TMEM33 enhances the SVCV-induced K48-linked ubiquitination of MAVS. EPC cells were transfected with 5 μg MAVS-Myc, 5 μg TMEM33-HA or empty vector, and 1 μg Ub-HA, Ub-K48O-HA or Ub-K63O-HA. At 24 h post-transfection, the cells were uninfected or infected with SVCV (MOI = 1) for 18 h, then the cells were treated with MG132 for 6 h. Cell lysates were IP with anti-Myc-affinity gel. Then the immunoprecipitates and WCLs were analyzed by IB with the indicated Abs. (F-J) Overexpression of TMEM33 inhibits the expression of *ifn* (F), *vig1* (G), *isg15-1* (H), *irf7* (I), and *rig-i* (J) induced by MAVS. EPC cells seeded in 6-well plates overnight were transfected with 2 μg of TMEM33-HA or empty vector together with 2 μg of MAVS-Myc or empty vector. At 24 h after transfection, total RNAs were extracted to examine the mRNA levels of cellular *ifn*, *vig1*, *isg15-1*, *irf7*, and *rig-i*. The relative transcriptional levels were normalized to the transcriptional level of the *β-actin* gene and were represented as fold induction relative to the transcriptional level in the control cells, which was set to 1. (K and L) TMEM33-ΔTM1 and TMEM33-ΔTM2 have no effect on MAVS-mediated suppression of viral genes transcription and protein expression. EPC cells were seeded in 6-well plates overnight and transfected with the indicated plasmids (2 μg each) for 24 h. At 24 h post-transfection, cells were infected with SVCV (MOI = 1) for 24 h. qPCR and immunoblot analysis were performed similarly as in C and D.

Given that TMEM33 targeted MAVS for Ub-proteasomal degradation, and fish MAVS is considered a strong antiviral factor, we investigated whether TMEM33 affects the cellular antiviral innate immunity mediated by MAVS. In antiviral effect evaluation assays, MAVS prevented CPE occurrence and decreased the viral titer 1150-fold compared to that in the control group. In contrast, simultaneous overexpression of TMEM33 increased the viral titer 240-fold compared to that in the MAVS group (Fig 8A and 8B). Moreover, TMEM33 significantly restored the transcription of viral genes and the expression of viral proteins in EPC cells inhibited by MAVS (Fig 8C and 8D). In addition, the ubiquitination analysis of MAVS in the TMEM33-overexpressed cells after SVCV infection was investigated. As shown in Fig 8E, SVCV infection induced MAVS ubiquitination with wild-type Ub and Ub-K48 but not with Ub-K63. And overexpression of TMEM33 significantly increased the SVCV-induced K48-lined ubiquitination of MAVS. In qPCR assays, the overexpression of TMEM33 inhibited the MAVS-induced expression of *ifn* and other ISG mRNAs (Fig 8F–8J). In addition, truncated mutant analysis showed that the viral genes transcription and protein expression was not influenced by TMEM33-ΔTM1 or TMEM33-ΔTM2 (Fig 8K and 8L). These results suggest that TMEM33 negatively regulates the MAVS-mediated cellular antiviral response and that the N-terminal TM1 and TM2 regions are necessary for this function.

### Zebrafish TMEM33 decreases TBK1 kinase activity in MITA and IRF3 phosphorylation

**Fig 9:**
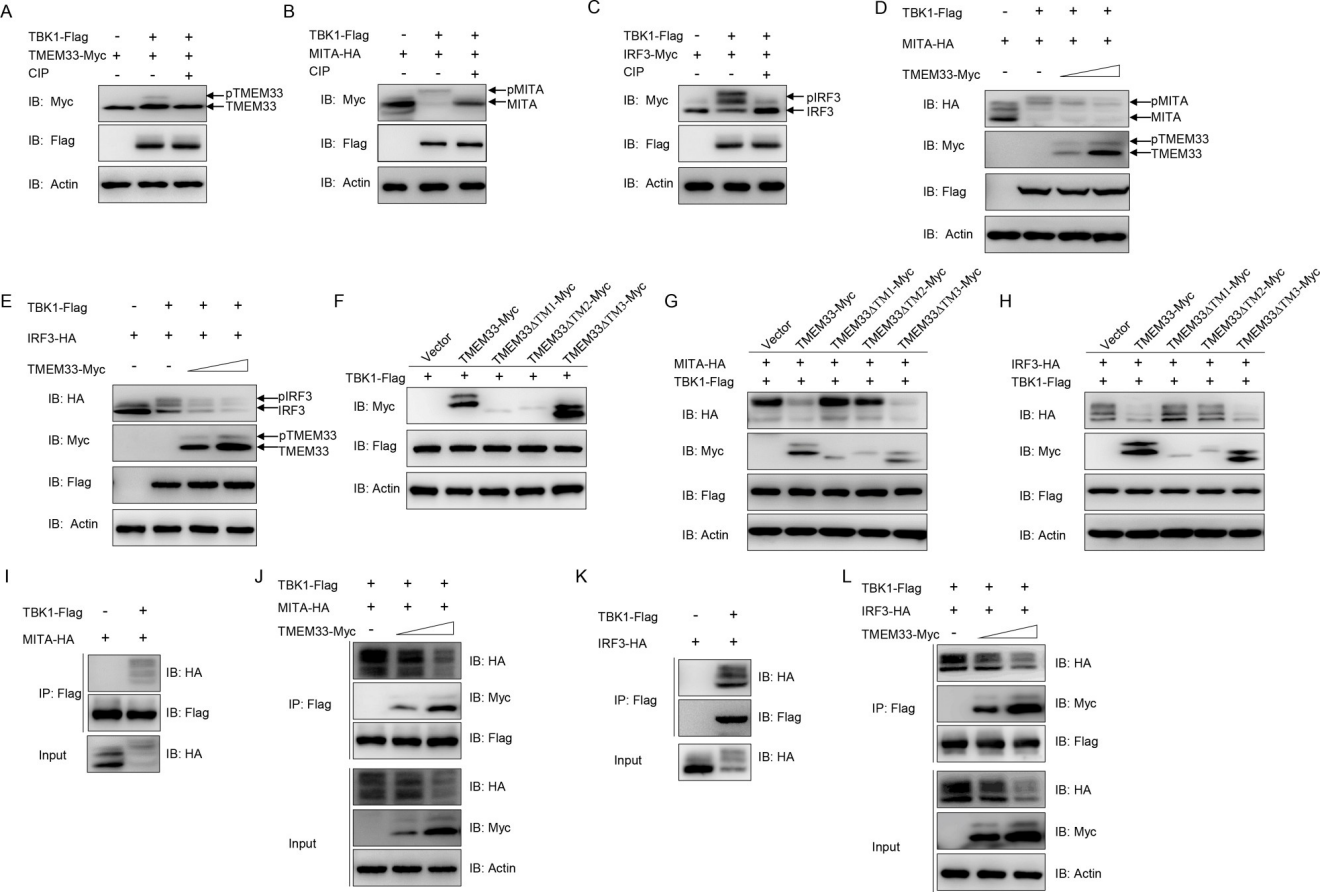
Zebrafish TMEM33 decreases TBK1-mediated MITA phosphorylation. (A) The amount of TBK1-phosphorylated TMEM33 is reduced by CIP treatment. EPC cells were seeded in 6-well plates overnight and transfected with the indicated plasmids (1 μg each) for 24 h. The cell lysates (100 μg) were treated with or without CIP (10 U) for 40 min at 37°C. Then the lysates were detected by IB with the indicated Abs. (B and C) TBK1 mediates the phosphorylation of MITA (B) and IRF3 (C). EPC cells were seeded into 6-well plates overnight and transfected with the indicated plasmids (1 μg each) for 24 h. The cell lysates (100 μg) were treated with or without CIP (10 U) for 40 min at 37°C. The lysates were then subjected to IB with the indicated Abs. (D and E) Overexpression of TMEM33 inhibits TBK1-mediated phosphorylation of MITA (D) and IRF3 (E) in a dose-dependent manner. EPC cells were seeded into 6-well plates overnight and co-transfected with 1 μg TBK1-Flag and 1 μg empty vector or TMEM33-Myc (0.5 and 1 μg, respectively), together with 1 μg MITA-HA or IRF3-HA for 24 h. The lysates were then subjected to IB with the indicated Abs. (F) TBK1 phosphorylates wild-type TMEM33 and TMEM33-ΔTM3. EPC cells were seeded in 6-well plates overnight and transfected with the indicated plasmids (1 μg each) for 24 h. Then the lysates were detected by IB with the indicated Abs. (G and H) TMEM33-ΔTM1 and TMEM33-ΔTM2 have no effect on TBK1-mediated phosphorylation of MITA (G) and IRF3 (H). EPC cells were seeded into 6-well plates overnight and co-transfected with 1 μg TBK1-Flag and 1 μg empty vector, TMEM33-Myc, TMEM33-ΔTM1-Myc, TMEM33-ΔTM2-Myc, or TMEM33-ΔTM3-Myc together with 1 μg MITA-HA or IRF3-HA for 24 h. The lysates were then subjected to IB with the indicated Abs. (I and J) TMEM33 blocks the interaction between TBK1 and MITA in a dose-dependent manner. EPC cells seeded in 10 cm^2^ dishes were co-transfected with 4 μg TBK1-Flag and 4 μg MITA-HA (I) or together with TMEM33-Myc (2 or 4 μg) (J). After 24 h, cell lysates were immunoprecipitated (IP) with anti-Flag affinity gel. Then the immunoprecipitates and WCLs were analyzed by IB with the indicated Abs. (J and K) TMEM33 disrupts the interaction between TBK1 and IRF3 in a dose-dependent manner. EPC cells seeded in 10 cm^2^ dishes were co-transfected with 4 μg TBK1-Flag and 4 μg IRF3-Myc (J) or together with TMEM33-HA (2 or 4 μg) (K) for 24 h. Immunoprecipitation and immunoblot analysis were performed similarly as in I and J. All experiments were repeated for at least three times with similar results.

We observed a high molecular form of TMEM33 when TBK1 was present (Fig 5A). TBK1 is known as a phosphokinase; therefore, we speculated that TMEM33 is phosphorylated by TBK1. When TMEM33 was co-transfected with TBK1, shifted bands with higher molecular weights were detected. These bands disappeared after treatment with CIP, demonstrating that fish TMEM33 is phosphorylated by TBK1 (Fig 9A). As substrates of TBK1 in mammals, fish MITA and IRF3 presented shifted bands when stimulated with TBK1 and the shift disappeared following CIP treatment, indicating that MITA and IRF3 were phosphorylated by TBK1 as well as in fish (Fig 9B and 9C). The processes of MITA/IRF3 phosphorylation by TBK1 are the classic signal transduction events in the RLR pathway. Since TMEM33 was also phosphorylated by TBK1, we subsequently investigated the role of TMEM33 in the TBK1 kinase activity. As shown in Fig 9D, MITA was phosphorylated by TBK1 and the phosphorylated MITA was gradually reduced by the overexpression of TMEM33 in a dose-dependent manner. Next, we investigated whether TMEM33 affects the phosphorylation of IRF3. As shown in Fig 9E, the IRF3 bands underwent an upward migration induced by TBK1, similar to MITA. The phosphorylation of IRF3 was also reduced dose-dependently with the overexpression of TMEM33. On the other hand, the TBK1-mediated phosphorylation of TMEM33 failed to occur in the TMEM33-ΔTM1 and TMEM33-ΔTM2 groups (Fig 9F). Accordingly, TMEM33-ΔTM1 and TMEM33-ΔTM2 did not affect the TBK1-induced phosphorylation of MITA and IRF3 (Fig 9G and 9H). It has been reported that MITA serves as a scaffold protein that associates with TBK1, facilitating IRF3 phosphorylation by TBK1 [9]. The above results suggest that TMEM33 reduces IRF3 phosphorylation; thus, we speculated that TMEM33 plays a role in regulating the MITA-TBK1 interactions. As shown in Fig 9I, MITA and TBK1 showed significant associations. As anticipated, this interaction was disrupted by TMEM33 in a dose-dependent manner (Fig 9J). Consistently, the interaction between TBK1 and IRF3 was also antagonized by TMEM33 (Fig 9K and 9L). Taken together, these data demonstrate that TMEM33 reduces the TBK1-induced phosphorylation of MITA/IRF3 through competitive phosphorylation by TBK1, impairing the interaction between MITA/IRF3 and TBK1.

### Zebrafish TMEM33 dampens TBK1-induced cellular antiviral response

**Fig 10:**
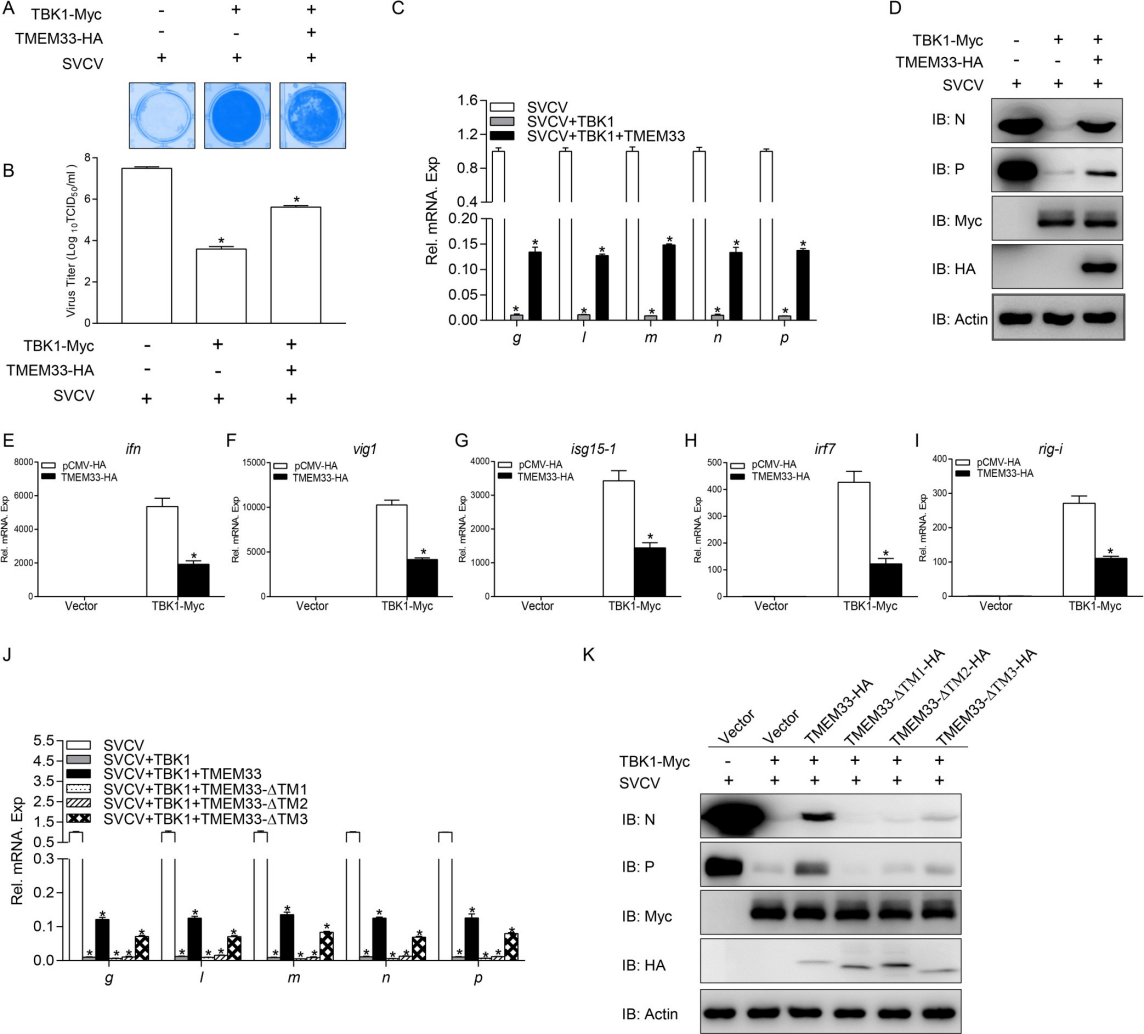
Zebrafish TMEM33 dampens TBK1-mediated cellular antiviral response. (A and B) Overexpression of TMEM33 increases TBK1-mediated decline of viral titer. EPC cells seeded in 24-well plates overnight were transfected with 0.25 μg of TBK1-Myc and 0.25 μg of TMEM33-HA or empty vector. At 24 h post-transfection, cells were infected with SVCV (MOI = 0.001) for 48 h. Then, cells were fixed with 4% PFA and stained with 1% crystal violet (A). Culture supernatants from the cells infected with SVCV were collected, and the viral titer was measured according to the method of Reed and Muench (B). (C) EPC cells seeded in 6-well plates overnight were transfected with 2 μg of TMEM33-HA or empty vector together with 2 μg of TBK1-Myc or empty vector. At 24 h post-transfection, cells were infected with SVCV (MOI = 1). After 24 h-infection, total RNAs were extracted to examine the mRNA levels of cellular *g*, *l*, *m*, *n*, and *p*. The relative transcriptional levels were normalized to the transcriptional level of the *β-actin* gene and were represented as fold induction relative to the transcriptional level in the control cells, which was set to 1. Data were expressed as mean ± SEM, n = 3. Asterisks indicate significant differences from control values (*, *p* < 0.05). (D) The same samples were prepared similarly as described above for panel C. The lysates were detected by IB with the indicated Abs. (E-I) Overexpression of TMEM33 blocks the expression of *ifn* (E), *vig1* (F), *isg15-1* (G), *irf7* (H), and *rig-i* (I) induced by TBK1. EPC cells seeded in 6-well plates overnight were transfected with 2 μg of TMEM33-HA or empty vector together with 2 μg of TBK1-Myc or empty vector. At 24 h after transfection, total RNAs were extracted to examine the mRNA levels of cellular *ifn*, *vig1*, *isg15-1*, *irf7*, and *rig-i*. The relative transcriptional levels were normalized to the transcriptional level of the *β-actin* gene and were represented as fold induction relative to the transcriptional level in the control cells, which was set to 1. (J and K) TMEM33-ΔTM1 and TMEM33-ΔTM2 have a little impact on TBK1-mediated inhibition of viral genes transcription and protein expression. EPC cells were seeded in 6-well plates overnight and transfected with the indicated plasmids (2 μg each) for 24 h. At 24 h post-transfection, cells were infected with SVCV (MOI = 1) for 24 h. qPCR and immunoblot analysis were performed similarly as in C and D. All experiments were repeated for at least three times with similar results.

Given that TMEM33 reduces the TBK1 kinase activity and fish TBK1 plays a vital role in defending against virus infections, the modulation of TMEM33 regulation of the TBK1-mediated cellular antiviral immune response was evaluated. First, we found that the viral titer decreased about 7200-fold in the TBK1 group compared to that in the control vector group, whereas TMEM33 increased the viral titer about 100-fold compared to that in only TBK1-expressing cells (Fig 10A and 10B). Moreover, the transcription and protein expression of SVCV-related genes which were suppressed by TBK1 were restored by TMEM33 in varying degrees (Fig 10C and 10D). In addition, TMEM33 suppressed the TBK1-induced transcription of *ifn* and related ISG mRNAs (Fig 10E and 10I). Finally, TMEM33-ΔTM1 and TMEM33-ΔTM2 had little impact on the TBK1-mediated inhibition of transcription and the protein expression of viral genes (Fig 10J and 10K). These data demonstrate that TMEM33 down-regulates the TBK1-mediated cellular antiviral immune response.

### The N-terminal TM1 and TM2 regions of TMEM33 are required for IFN inhibitory activity

**Fig 11:**
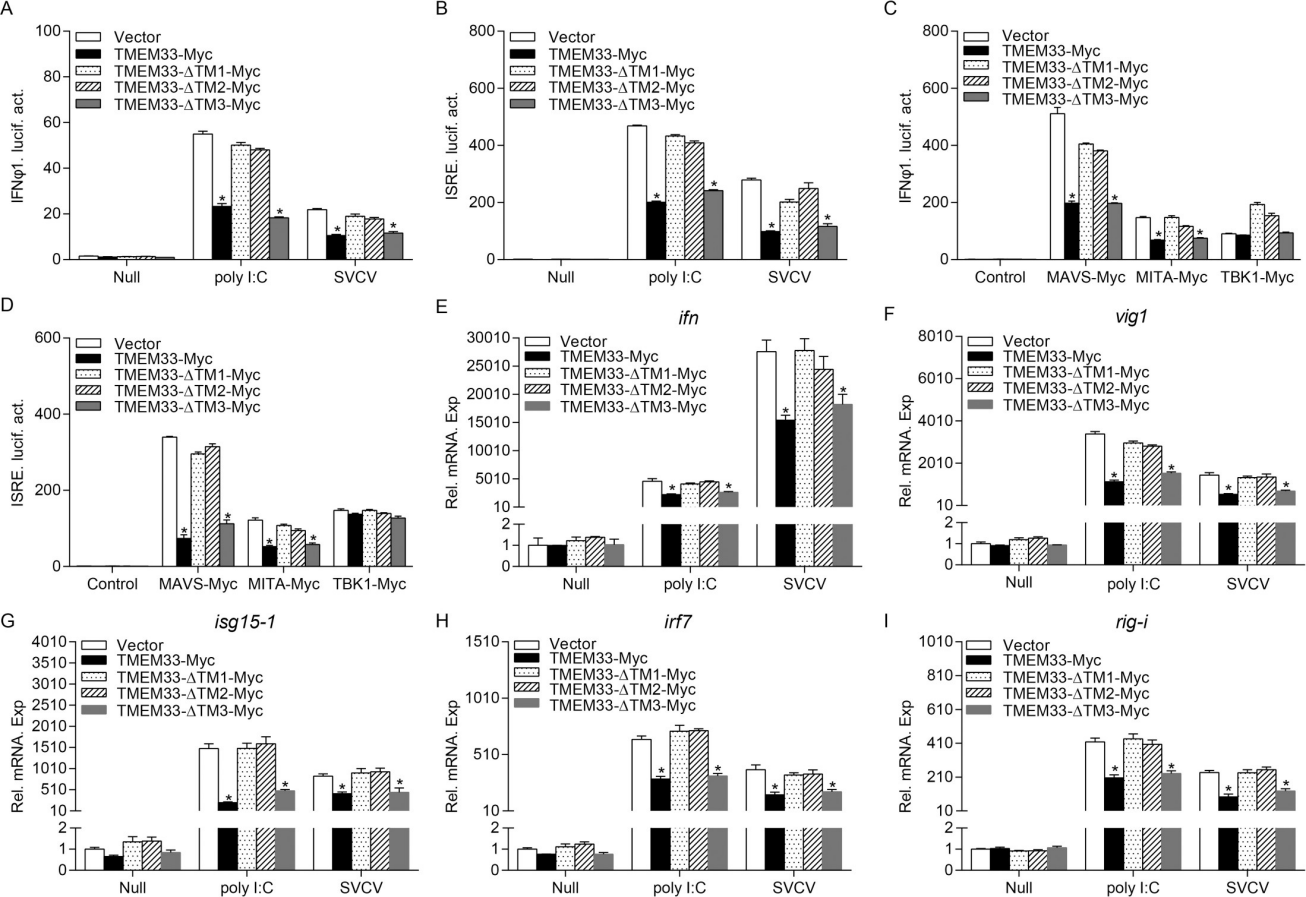
The N-terminal TM1 and TM2 regions of TMEM33 are essential for its inhibitory activity. (A and B) TMEM33-ΔTM1 and TMEM33-ΔTM2 have no effect on poly I:C/SVCV-induced IFNφ1pro/ISRE-Luc activation. EPC cells were seeded in 24-well plates and transfected the next day with 250 ng IFNφ1pro-Luc (A) or ISRE-Luc (B) and 25 ng pRL-TK, plus 250 ng TMEM33-Myc, TMEM33-ΔTM1-Myc, TMEM33-ΔTM2-Myc, TMEM33-ΔTM3-Myc, or pCMV-Myc (control vector). At 24 h post-transfection, cells were untreated (null) or transfected with poly I:C (1 μg/ml) or treated with SVCV (MOI = 1). Luciferase activities were monitored at 24 h after stimulation. The promoter activity is presented as relative light units (RLU) normalized to *Renilla* luciferase activity. (C and D) TMEM33-ΔTM1 and TMEM33-ΔTM2 have no effect on RLRs-induced IFNφ1pro/ISRE-Luc activation. EPC cells were seeded in 24-well plates and transfected the next day with 250 ng IFNφ1pro-Luc (C) or ISRE-Luc (D) and 250 ng MAVS-Myc, MITA-Myc, or TBK1-Myc and 25 ng pRL-TK, plus 250 ng TMEM33-Myc, TMEM33-ΔTM1-Myc, TMEM33-ΔTM2-Myc, TMEM33-ΔTM3-Myc, or pCMV-Myc (control vector). Luciferase activities were monitored at 24 h after transfection. (E-I) TMEM33-ΔTM1 and TMEM33-ΔTM2 have no effect on poly I:C/SVCV-induced the expression of *ifn* (E), *vig1* (F), *isg15-1* (G), *irf7* (H), and *rig-i* (I). EPC cells seeded in 6-well plates overnight were transfected with the indicated plasmids (2 μg each) for 24 h. At 24 h post-transfection, cells were untreated (null) or transfected with poly I:C (1 μg/ml) or stimulated with SVCV (MOI = 1) for 24 h. Total RNAs were extracted to examine the mRNA levels of cellular *ifn*, *vig1*, *isg15-1*, *irf7*, and *rig-i*. The relative transcriptional levels were normalized to the transcription of *β-actin* and represented as fold induction relative to the transcriptional level in the control cells, which was set to 1. Data were expressed as mean ± SEM, *n* = 3. Asterisks indicate significant differences from control (*, *p* < 0.05).

The above results demonstrated that the N-terminal TM1 and TM2 regions of TMEM33 are required and sufficient for the modulation of MAVS and TBK1. Therefore, we investigated whether these regions in TMEM33 are responsible for suppressing IFN production. First, a luciferase assay suggested that wild-type TMEM33 and TMEM33-ΔTM3 inhibited the activation of IFNφ1pro or ISRE induced by poly I:C or SVCV. TMEM33-ΔTM1 and TMEM33-ΔTM2 had no effect on this inhibition (Fig 11A and 11B). In the RLR signaling pathways, the induction of IFNφ1pro or ISRE by MAVS and MITA was unchanged with the overexpression of TMEM33-ΔTM1 or TMEM33-ΔTM2, as anticipated (Fig 11C and 11D). At the mRNA level, TMEM33-ΔTM1 and TMEM33-ΔTM2 did not repress the poly I:C or SVCV-induced expression of *ifn* and several other ISG mRNAs (Fig 11E and 11I). Collectively, these data suggest that the N-terminal TM1 and TM2 regions are essential for the IFN inhibition of TMEM33.

## Discussion

Viral infection leads to the transcriptional induction of IFNs and other cytokines that have inhibitory effects on virus replication in infected cells [52]. However, uncontrolled excessive production of IFNs causes significant chronic inflammation and autoimmune diseases [53]. A multitude of studies suggest that host cells adopt at least two distinct strategies to ensure the proper production of IFNs and to return to a homeostatic state following viral infection [30]. One of the mechanisms is posttranslational modification (PTM) of signaling molecules, such as ubiquitination, dephosphorylation, sumoylation, and methylation. For instance, several members of the Ub ligase (E3) family have been implicated in the inhibition of IFN production by targeting the key components of RLR signaling pathways for degradation, such as RING finger protein 125 (RNF125), atrophin-1-interacting protein 4 (AIP4), and RTA-associated Ub ligase (RAUL) [54–56]. Second, some inhibitory proteins, such as Polo-like kinase 1 (PLK1), ISG15, and suppressor of IKKε (SIKE), physically associate with key molecules in the RLR pathways to sequester them in inactive forms [57–59]. In the present work, we found that transmembrane protein TMEM33 degrades MAVS in a K48-linked Ub-proteasome-dependent manner and reduces MITA/IRF3 phosphorylation by acting as a decoy substrate of TBK1, thereby preventing IFN expression.

Ubiquitination is a vital PTM in the control of RLR pathway activity that regulates the stability, localization, and activity of target proteins [60]. This process involves three classes of enzymes: Ub-activating enzyme (E1), Ub-conjugating enzyme (E2), and E3s [61]. E3s determine the substrate specificity and are categorized into two classes: homologous to the E6-associated protein C terminus (HECT) domain E3s and RING or RING-like (such as, U-box or PHD) domain E3s [62]. In mammals, several E3 ligases, including AIP4, Smurf2, and tripartite motif containing 25 (TRIM25), have been reported to weaken host immune responses through catalyzing the K48-linked polyubiquitination and degradation of MAVS [35, 55, 63]. In addition, some molecules have been shown to mediate the degradation of MAVS through recruiting E3 ligases, for instance, poly (rC) binding protein 2 (PCBP2) and Nedd4 family interacting protein 1 (Ndfip1) [55, 64]. In fish, several molecules, such as grass carp LGP2 and zebrafish N-myc downstream-regulated gene 1a (NDRG1a) have been identified as negative regulators of RLR-mediated IFN production by regulating their ubiquitination [65, 66]. A BLASTP search revealed that zebrafish TMEM33 protein lacks a classical E3s domain. Hence, further research should determine whether TMEM33 acts as a new unknown E3 ligase or recruits another E3 ligase to catalyze the K48-linked ubiquitination of MAVS.

Upon virus infection, MAVS forms prion-like aggregates on the mitochondria and acts as a scaffold to recruit downstream/upstream proteins, leading to the production of IFNs [67]. Therefore, the activity of MAVS must be tightly regulated to maintain immune balance. MAVS is regulated by PTMs such as phosphorylation and ubiquitination. It has been reported that tyrosine kinase c-Abl and Nemo-like kinase (NLK) can modulate MAVS activity by phosphorylating MAVS [68, 69]. TRIM31 catalyzes the K63-linked polyubiquitination of MAVS and promotes its activation [70]. Ndfip1 and von Hippel-Lindau (VHL) promote K48-linked polyubiquitination of MAVS, which leads to its degradation [64, 71]. In this study, TMEM33 interacted with MAVS and facilitated the K48-linked ubiquitination and degradation of fish MAVS. Thus, our study provides evidence that the negative regulatory mechanisms of MAVS are conserved in lower vertebrates.

As a critical kinase involved in multiple signaling pathways, TBK1 activity must be tightly modulated to maintain immune homeostasis in a variety of ways, such as phosphorylation/dephosphorylation, ubiquitination/deubiquitination, kinase activity regulation, and prevention of functional TBK1-containing complex formation [72]. In mammals, numerous molecules have been identified as TBK1-negative modulators. In fish, there are few reports about the mechanisms underlying the negative regulation of TBK1. In this study, we found that zebrafish TMEM33 negatively regulated IFN induction by decreasing TBK1-mediated MITA/IRF3 phosphorylation. It has been reported that the formation of functional TBK1-complexes is necessary for the TBK1 activity in antiviral immune responses. Thus, preventing the formation of functional TBK1-complexes is the main mechanism for the inhibition of IFN production. For example, nuclear receptor estrogen-related receptor α (ERRα) was found to inhibit IFN production by impeding the formation of the TBK1-IRF3 complex [73]. Our results demonstrated that TMEM33 was competitively phosphorylated by TBK1 and disrupted the interactions of TBK1 with MITA and IRF3, which resulted in the suppression of MITA/IRF3 phosphorylation and IFN production.

TMEMs are proteins that span biological membranes. Many of them are located at the membranes of organelles, including mitochondria, ER, lysosome, and Golgi [74]. TMEMs are present in many cell types and participate in a variety of physiological functions such as immune responses (TMEM173) [75], smooth muscle contractions (TMEM16) [76], and cancer (TMEM45A) [77]. Previous studies revealed that the TMEM33 family, which is conserved in yeasts and mammals, has three TMs, is localized in the ER, and regulates the tubular structure of the ER [78]. In this study, zebrafish TMEM33, which is also localized in the ER, was identified. Therefore, the binding between TMEM33 and reticulons is likely to be evolutionarily conserved, raising the possibility that TMEM33 is an evolutionarily conserved regulator for reticulons. In addition, it has been reported that upon stimulation, MITA is translocated from the ER to the cytoplasmic punctate structures to assemble with TBK1, and this dynamic traffic is essential to activate the innate immune response [79]. The ER-localized TMEM33 may retain MITA at the ER to block the interactions of MITA with TBK1. Further studies will be required to address these concerns.

**Fig 12:**
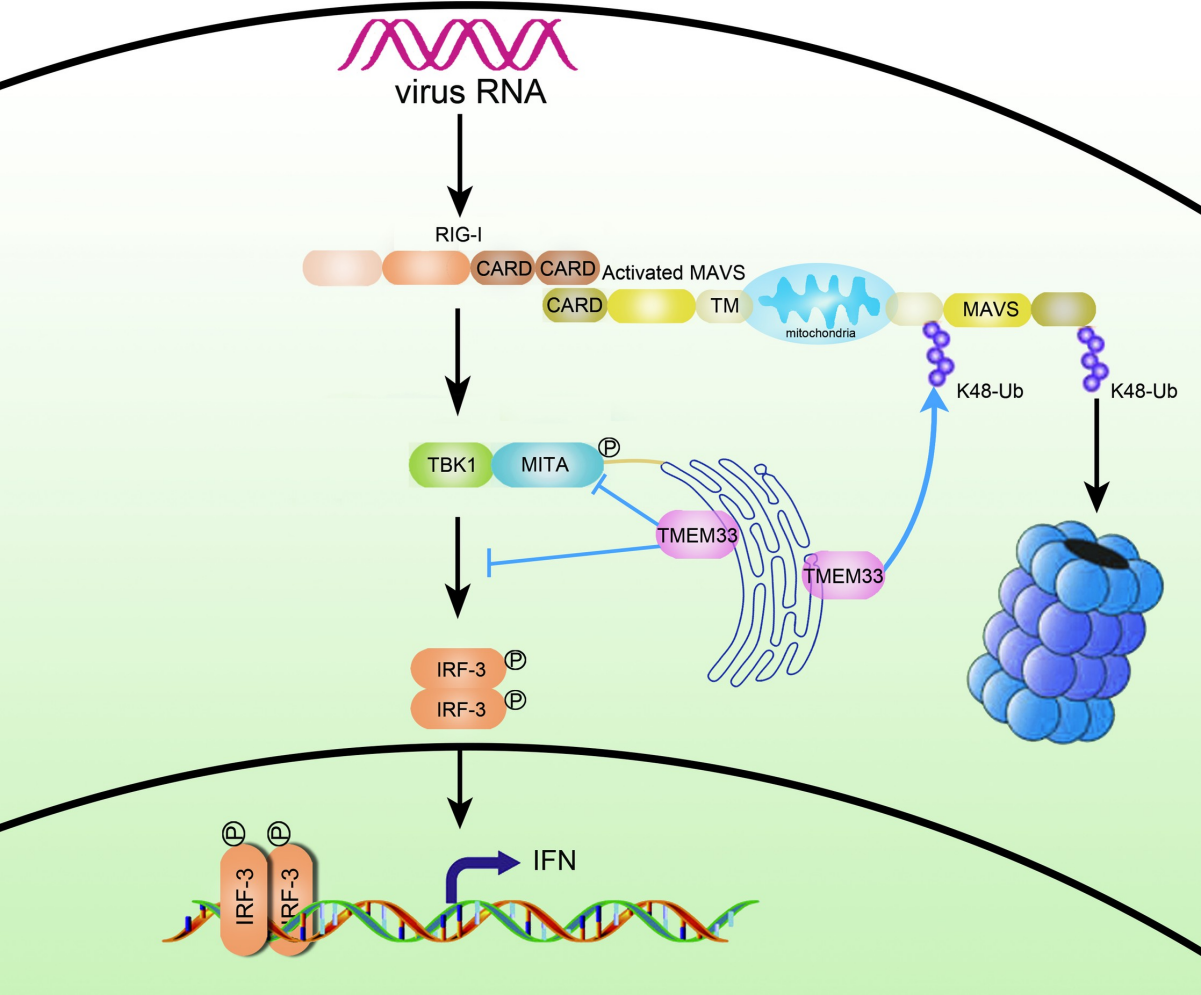
A model on the dual roles of zebrafish TMEM33 in negative regulation of RLR-mediated signaling pathways. Upon virus infection, fish RIG-I senses the viral RNA and interacts with MAVS, leading to the recruitment of TBK1, which phosphorylates MITA and IRF3, then induces IFN production. TMEM33, an ER-localized protein, triggers the K48-linked ubiquitination and degradation of MAVS, meanwhile, it also reduces MITA/IRF3 phosphorylation by acting as a substrate of TBK1, thereby preventing IFN expression.

In summary, we proposed a working model on the dual functions of TMEM33 in negatively regulating RLR-mediated IFN production (Fig 12). TMEM33, an ER-localized protein, serves as a negative regulator of IFN production by inducing the Ub-proteasomal degradation of MAVS and reducing the phosphorylation of MITA/IRF3 via acting as a decoy substrate of TBK1. Our results reveal a previously unreported role for fish TMEM33 in regulating cellular immune responses and provide important evidence that the conserved negative regulatory mechanisms involved in antiviral immunity also exist in teleost.

## Methods

### Ethics statement

This study is in compliance with all ethical regulations and was approved by the Committee on the Ethics of Animal Experiments of the Institute of Hydrobiology, Chinese Academy of Sciences (No. 2019–040).

### Cells and viruses

Human embryonic kidney (HEK) 293T cells were provided by Dr. Xing Liu (Institute of Hydrobiology, Chinese Academy of Sciences) and were grown at 37°C in 5% CO_2_ in Dulbecco’s modified Eagle’s medium (DMEM; Invitrogen) supplemented with 10% fetal bovine serum (FBS, Invitrogen). Zebrafish liver (ZFL) cells (American Type Culture Collection, ATCC) were cultured at 28°C in 5% CO_2_ in Ham’s F12 nutrient mixture medium (Invitrogen) supplemented with 10% FBS. Epithelioma papulosum cyprini (EPC) cells were obtained from China Center for Type Culture Collection (CCTCC) and were maintained at 28°C in 5% CO_2_ in medium 199 (Invitrogen) supplemented with 10% FBS. Spring viremia of carp virus (SVCV), a negative ssRNA virus, was propagated in EPC cells at 28°C until a cytopathogenic effect (CPE) was observed; then the harvested cell culture fluid containing SVCV (multiplicity of infection (MOI) = 1000) was centrifuged at 4 × 10^3^ *g* for 20 min to remove the cell debris, and the supernatant was stored at -80°C until used.

### Plasmid construction and reagents

The sequence of zebrafish TMEM33 (GenBank accession number NM_213663.1) was obtained from the NCBI (National Center for Biotechnology Information) website (http://www.ncbi.nlm.nih.gov/). Using the cDNA of the tissues from adult zebrafish as template, the open reading frame (ORF) of zebrafish TMEM33 and the truncated mutants of TMEM33 were amplified by polymerase chain reaction (PCR) and cloned into pcDNA3.1(+) (Invitrogen), pCMV-Myc (Clontech), or pCMV-HA vectors (Clontech), respectively. The ORFs of zebrafish MAVS (NM_001080584.2), MAVS mutants, MITA (NM_001278837.1), MITA mutants, TBK1 (NM_001044748.2), TBK1 mutants, and IRF3 (NM_001143904) were also subcloned into pcDNA3.1(+), pCMV-Myc, pCMV-HA, and pCMV-Tag2C vectors (Clontech), respectively. For subcellular localization, the ORF of zebrafish TMEM33 was inserted into pEGFP-N3 vector (Clontech). The ORFs of MAVS, MITA, TBK1, and IRF3 were also inserted into pDsRed-N1 vector (Clontech). The pDsRed-ER plasmid was purchased from Clontech. The plasmids containing zebrafish IFNφ1pro-Luc and ISRE-Luc in the pGL3-Basic luciferase reporter vector (Promega) were constructed as described previously [41]. The *Renilla* luciferase internal control vector (pRL-TK) was purchased from Promega. Ub-K48O-HA and Ub-K63O-HA were expression plasmids for HA-tagged Lys-48- and Lys-63-only ubiquitin mutants (all lysine residues except Lys-48 or Lys-63 are mutated). All constructs were confirmed by DNA sequencing. The primers including the restriction enzyme cutting sites used for plasmid construction are listed in S1 Table. MG132 and polyinosinic-polycytidylic acid (poly I:C) were purchased from Sigma-Aldrich and used at a final concentration of 20 μM/ml and 1 μg/ml, respectively.

### Luciferase activity assay

EPC cells were seeded in 24-well plates overnight and co-transfected with the indicated luciferase reporter plasmids and expression vectors. The empty vector pcDNA3.1(+) was used to ensure equivalent amounts of total DNA in each well. Transfection of poly I:C by using FishTrans (MeiSenTe Biotechnology) was performed at 24 h before cell harvest. At 48 h post-transfection, the cells were washed with phosphate-buffered saline (PBS) and lysed for measuring luciferase activity by the Dual-Luciferase Reporter Assay System (Promega) according to the manufacturer’s instructions. Firefly luciferase activity was normalized on the basis of *Renilla* luciferase activity.

### Transient transfection and virus infection

Transient transfections were performed in EPC cells seeded in 6-well or 24-well plates or in ZFL cells seeded in 6-well plates by using FishTrans (MeiSenTe Biotechnology) according to the manufacturer’s protocol. For the antiviral assay using 24-well plates, EPC cells were transfected with 0.5 μg TMEM33-Myc or the empty vector. At 24 h post-transfection, cells were infected with SVCV (MOI = 0.001). After 48 h or 72 h, supernatant aliquots were harvested for detection of virus titers, the cell monolayers were fixed by 4% paraformaldehyde (PFA) and stained with 1% crystal violet for visualizing CPE. For virus titration, 200 μl of culture medium were collected at 48 h post-infection and used for detection of virus titers according to the method of Reed and Muench. The supernatants were subjected to 3-fold serial dilutions and then added (100 μl) onto a monolayer of EPC cells cultured in a 96-well plate. After 48 or 72 h, the medium was removed and the cells were washed with PBS, fixed by 4% PFA and stained with 1% crystal violet. The virus titer was expressed as 50% tissue culture infective dose (TCID_50_/ml).

### RNAi experiments

EPC cells were seeded in 6-well plates overnight and transfected with 100 nM siRNA of TMEM33 or the negative control (si-Nc) by using FishTrans (MeiSenTe Biotechnology). Small interfering RNAs (siRNA) of TMEM33 and si-Nc were obtained from GenePharma (Shanghai, China). The following sequences were targeted for EPC TMEM33, respectively: si-TMEM33#1: GCUGCCACUAUCUGCUCUATT; si-TMEM33#2: GCCUCAUGUUUGUGAGGAATT.

### RNA extraction, reverse transcription, and quantitative PCR (qPCR)

Total RNAs were extracted by the TRIzol reagent (Invitrogen). Genomic DNA was thoroughly digested by RNase free DNase (Promega). First-strand cDNA was synthesized by using a GoScript reverse transcription system (Promega) according to the manufacturer’s instructions. qPCR was performed with Fast SYBR green PCR master mix (Bio-Rad) on the CFX96 real-time system (Bio-Rad). PCR conditions were as follows: 95°C for 5 min and then 40 cycles of 95°C for 20 s, 60°C for 20 s, and 72°C for 20 s. All primers used for qPCRs are shown in S1 Table, and *β-actin* gene was used as an internal control. The relative fold changes were calculated by comparison to the corresponding controls using the 2^-ΔΔCt^ method. Three independent experiments were conducted for statistical analysis.

### Co-immunoprecipitation (Co-IP) assay

For Co-IP experiments, HEK 293T cells were used instead of EPC cells (transfection efficiency approximately 60%) due to the superhigh transfection efficiency of HEK 293T cells (90%). Cells seeded in 10 cm^2^ dishes overnight were transfected with a total of 10 μg of the plasmids indicated on the figures. At 24 h post-transfection, the medium was removed carefully, and the cell monolayer was washed twice with 10 ml ice-cold PBS. Then the cells were lysed in 1 ml of radioimmunoprecipitation (RIPA) lysis buffer [1% NP-40, 50 mM Tris-HCl, pH 7.5, 150 mM NaCl, 1 mM EDTA, 1 mM NaF, 1 mM sodium orthovanadate (Na_3_VO_4_), 1 mM phenyl-methylsulfonyl fluoride (PMSF), 0.25% sodium deoxycholate] containing protease inhibitor cocktail (Sigma-Aldrich) at 4°C for 1 h on a rocker platform. The cellular debris was removed by centrifugation at 12,000 × *g* for 15 min at 4°C. The supernatant was transferred to a fresh tube and incubated with 30 μl anti-Flag/Myc affinity gel (Sigma-Aldrich) overnight at 4°C with constant agitation. These samples were further analyzed by immunoblotting (IB). Immunoprecipitated proteins were collected by centrifugation at 5000 × *g* for 1 min at 4°C, washed three times with lysis buffer and resuspended in 50 μl 2 × SDS sample buffer. The immunoprecipitates and whole cell lysates (WCLs) were analyzed by IB with the indicated antibodies (Abs).

### In vivo ubiquitination assay

Transfected EPC cells were washed twice with 10 ml ice-cold PBS and then digested with 1 ml 0.25% trypsin-EDTA (1×) (Invitrogen) for 2–3 min until the cells were dislodged. 200 μl FBS was added to neutralize the trypsin and the cells were resuspended into 1.5 ml centrifuge tube, centrifuged at 2000 × *g* for 5 min. The supernatant was discarded and the cell precipitations were resuspended using 1 ml PBS and centrifuged at 2000 × *g* for 5 min. The collected cell precipitations were lysed using 100 μl PBS containing 1% SDS and denatured by heating for 10 min. The supernatants were diluted with lysis buffer until the concentration of SDS was decreased to 0.1%. The diluted supernatants were incubated with 20 μl anti-Myc affinity gel overnight at 4°C with constant agitation. These samples were further analyzed by IB. Immunoprecipitated proteins were collected by centrifugation at 5000 × *g* for 1 min at 4°C, washed three times with lysis buffer and resuspended in 50 μl 2 × SDS sample buffer.

### Immunoblot analysis

Immunoprecipitates or WCLs were separated by 10% SDS-PAGE and transferred to polyvinylidene difluoride (PVDF) membrane (Bio-Rad). The membranes were blocked for 1 h at room temperature in TBST buffer (25 mM Tris-HCl, 150 mM NaCl, 0.1% Tween 20, pH 7.5) containing 5% nonfat dry milk, probed with the indicated primary Abs at an appropriate dilution overnight at 4°C, washed three times with TBST, and then incubated with secondary Abs for 1 h at room temperature. After three additional washes with TBST, the membranes were stained with the Immobilon Western chemiluminescent horseradish peroxidase (HRP) substrate (Millipore) and detected by using an ImageQuant LAS 4000 system (GE Healthcare). Abs were diluted as follows: anti-β-actin (Cell Signaling Technology) at 1:1,000, anti-Flag/HA (Sigma-Aldrich) at 1:3,000, anti-Myc (Santa Cruz Biotechnology) at 1:2,000, and HRP-conjugated anti-mouse/rabbit IgG (Thermo Scientific) at 1:5,000. Results are representative of three independent experiments.

### In vitro protein dephosphorylation assay

Transfected EPC cells were lysed as described above, except that the phosphatase inhibitors (Na_3_VO_4_ and EDTA) were omitted from the lysis buffer. Protein dephosphorylation was carried out in 100 μl reaction mixtures consisting of 100 μg of cell protein and 10 units (U) of calf intestinal phosphatase (CIP) (Sigma-Aldrich). The reaction mixtures containing 20 mM Tris, pH 7.5, 10 mM MgCl_2_, 5 mM MnCl_2_, 1 mM DTT, protease inhibitor and phosphorylated protein were incubated at 37°C for 40 min, followed by immunoblot analysis.

### Fluorescent microscopy

EPC cells were plated onto coverslips in 6-well plates and transfected with the plasmids indicated on the figures for 24 h. Then the cells were washed twice with PBS and fixed with 4% PFA for 1 h. After being washed three times with PBS, the cells were stained with 1 μg/ml 4′, 6-diamidino-2-phenylindole (DAPI; Beyotime) for 15 min in the dark at room temperature. Finally, the coverslips were washed and observed with a confocal microscope under a 63× oil immersion objective (SP8; Leica).

### Statistics analysis

Luciferase, qPCR, and virus titer detection data are expressed as mean ± standard error of the mean (SEM). The p values were calculated by the Student’s t-test. A *p* value < 0.05 was considered statistically significant.

## Supporting information

S1 Table Primers used in this study. (DOCX)
