# Supplementary material for: A novel role of Zebrafish TMEM33 in negative regulation of interferon production by two distinct mechanisms
Source: PLoS Pathog. 2021 Feb 18;17(2):e1009317. doi: 10.1371/journal.ppat.1009317 (PMC7891750; doi:10.1371/journal.ppat.1009317)
Supplement: S1 Table — (DOCX) [file ppat.1009317.s001.docx]

S1 Table. Primers used in this study.

| **Name** | **Sequence (5’→3’)** | **Purpose** |
| --- | --- | --- |
| pcDNA3.1/pCMV-HA/Myc-TMEM33(NM_213663.1)-F | CCGGAATTCCGATGGCAGACACCGAGCAAAG | Eukaryotic expression |
| pcDNA3.1/pCMV-HA/Myc-TMEM33-R | CCGCTCGAGTTAGACGCCGGTTGGAGCG |  |
| DsRed-MAVS(NM_001080584.2)-F | CGCGGATCCGCCACCATGTCACTGACACGTGAGC |  |
| DsRed-MAVS-R | CCCATCGATATGATTGAGCTTCCAG |  |
| DsRed-TBK1(NM_001044748.2)-F | CGCGGATCCGCCACCATGCAGAGTACGGCCAAT |  |
| DsRed-TBK1-R | CCGGAATTCCGCATCCGCTCCACTG |  |
| DsRed-MITA(NM_001278837.1)-F | CGCGGATCCGCCACCATGTCTGTGATGGGAG |  |
| DsRed-MITA-R | CCGGAATTCCGGTTTTGTTTCATTGC |  |
| DsRed-IRF3(NM_001143904)-F | CCCATCGATGCCACCATGACTCAAGCAAAACCG |  |
| DsRed-IRF3-R | CCGGAATTCCGGCAGAGCTCCATCA |  |
| pEGFP-N3-TMEM33(NM_213663.1)-F | CTAGCTAGCATGGCAGACACCGAGCAAAG |  |
| pEGFP-N3-TMEM33(NM_213663.1)-R | CGCGGATCCGACGCCGGTTGGAGCG |  |
| pCMV-Myc/Tag2C-MAVS-F | CCGCTCGAGCGATGTCACTGACACGTGAGC |  |
| pCMV-Myc/Tag2C-MAVS-R | ATTTGCGGCCGCTTAATGATTGAGCTTCCAG |  |
| pCMV-Myc/Tag2C-TBK1-F | CGCGGATCCCGATGCAGAGTACGGCCAAT |  |
| pCMV-Myc/Tag2C-TBK1-R | AACTCGAGTCACATCCGCTCCACTG |  |
| pCMV-Myc/Tag2C-MITA-F |  |  |
| pCMV-Myc/Tag2C-MITA-R | AACTCGAGTTAGTTTTGTTTCATTGC |  |
| pCMV-Myc/Tag2C-IRF3-F | CCGGAATTCCGATGACTCAAGCAAAACCG |  |
| pCMV-Myc/Tag2C-IRF3-R | AACTCGAGTTAGCAGAGCTCCATCA |  |
| *Drtmem33-*F | CCACTATCTGCTCTACTCCCTC | Real-time PCR |
| *Drtmem33*-R | GTGTATGTAGTGGCGTGGAGAAGTG |  |
| *Drifn1*-F | GAATGGCTTGGCCGATACAGGATA |  |
| *Drifn1*-R | TCCTCCACCTTTGACTTGTCCATC |  |
| *Drirf7*-F | CCTTGGTGACGCGGATGT |  |
| *Drirf7*-R | GGAAGTTTGTCTTCCATTTTGCTTT |  |
| *Drβ-actin*-F | CCGTGACATCAAGGAGAAGCT |  |
| *Drβ-actin*-R | TCGTGGATACCGCAAGATTCC |  |
| *mavs-epc*-F | GAATGTCCCTGTCCGAGAAA |  |
| *mavs-epc*-R | TCTGAACATGCTCGTTTGCAG |  |
| *ifn-epc*-F | ATGAAAACTCAAATGTGGACGTA |  |
| *ifn-epc*-R | GATAGTTTCCACCCTTTCCTTAA |  |
| *vig1-epc*-F | AGCGAGGCTTACGACTTCTG |  |
| *vig1-epc*-R | GCACCAACTCTCCCAGAAAA |  |
| *isg15-epc*-F | CAGCCTTGAGGATGATTCCAG |  |
| *isg15-epc*-R | TGCCGTTGTAAATCAGTCG |  |
| *irf7-epc*-F | GGAGGACCAACACAAAGTCTATC |  |
| *irf7-epc*-R | CATTTCCTCCACTTGGCTGAG |  |
| *rig-i-epc-*F | TGCTGGACCGGATGTGTTATCT |  |
| *rig-i-epc*-R | TGGTGATCGATGGTTCGATTCT |  |
| *tmem33-epc*-F | CCACTATCTCCTCTACTCCCTC |  |
| *tmem33-epc*-R | GTATAGGTGG TGGCGTGGAG AAGTG |  |
| *β-actin-epc*-F | CACTGTGCCCATCTACGAG |  |
| *β-actin-epc*-R | CCATCTCCTGCTCGAAGT |  |
